# Supplementary material for: Immunoinformatics Identification of the Conserved and Cross-Reactive T-Cell Epitopes of SARS-CoV-2 with Human Common Cold Coronaviruses, SARS-CoV, MERS-CoV and Live Attenuated Vaccines Presented by HLA Alleles of Indonesian Population
Source: Viruses. 2022 Oct 24;14(11):2328. doi: 10.3390/v14112328 (PMC9699331; doi:10.3390/v14112328)
Supplement: Supplementary file 1 [file viruses-14-02328-s001.zip › Table S1 and S2.pdf]

Supplementary Materials

Table S1. Full list of predicted 9-mer CTL epitopes from five different SARS-CoV-2 proteins (ORF1ab, S, E, M, and N) with %Rank less than 1% identified from NetCTLpan 1.1 along with their immunogenicity score. A total of 414 promiscuous peptides (bind to more than one different HLA alleles) out of 1,599 predicted peptides were identified and indicated by green color highlight. A total of 1,299 peptides with positive immunogenicity scores are identified and highlighted by bold-colored text.

Protein	Start Residue	Peptide	HLA Class I Allele	Immunogenicity
ORF1ab	2	SLVPGFNEK	HLA-A*11:01	**0.08077**
	44	HLKDGTCGL	HLA-A*02:03	**0.40234**
	64	EQPYVFIKR	HLA-A*33:03	**0.07638**
	83	VMVELVAEL	HLA-A*02:01, HLA-A*02:03	**0.22188**
	109	HVGEIPVAY	HLA-B*15:02, HLA-B*15:21, HLA-B*35:05	-0.24214
	115	VAYRKVLLR	HLA-A*33:03	**0.08565**
	134	SYGADLKSF	HLA-A*24:02, HLA-A*24:07	**0.29037**
	140	KSFDLGDEL	HLA-B*58:01	**0.10192**
	175	ELNGGAYTR	HLA-A*33:03	**0.04585**
	181	YTRYVDNNF	HLA-B*15:02, HLA-B*15:13, HLA-B*35:05	**0.17504**
	231	REHEHEIAW	HLA-B*18:01, HLA-B*44:03	-0.01938
	246	KSYELQTPF	HLA-B*15:02, HLA-B*15:13, HLA-B*15:21, HLA-B*58:01	**0.05549**
	248	YELQTPFEI	HLA-B*18:01, HLA-B*38:02, HLA-B*44:03	**0.05473**
	250	LQTPFEIKL	HLA-B*38:02	**0.11198**
	254	FEIKLAKKF	HLA-B*18:01, HLA-B*44:03	**0.01738**
	257	KLAKKFDTF	HLA-A*24:02, HLA-A*24:07	-0.05331
	278	SIIKTIQPR	HLA-A*33:03, HLA-A*34:01	-0.24371
	281	KTIQPRVEK	HLA-A*11:01	**0.21515**
	295	FMGRIRSVY	HLA-B*15:02, HLA-B*15:13, HLA-B*15:21, HLA-B*35:05	**0.23703**
	312	NQMCLSTLM	HLA-B*15:02, HLA-B*15:21, HLA-B*38:02	**0.11957**
	313	QMCLSTLMK	HLA-A*11:01	-0.10819
	340	CEFCGTENL	HLA-B*18:01, HLA-B*38:02, HLA-B*44:03	-0.0585
	350	KEGATTCGY	HLA-B*44:03	-0.03267
	375	SEVGPEHSL	HLA-B*18:01, HLA-B*38:02, HLA-B*44:03	-0.17836
	388	NESGLKTIL	HLA-B*18:01, HLA-B*38:02	**0.04268**
	402	TIAFGGCVF	HLA-B*15:02, HLA-B*15:21	-0.29177
	410	FSYVGCHNK	HLA-A*11:01	**0.14136**
	444	GLNDNLLEI	HLA-A*02:01, HLA-A*02:03	**0.2593**
	467	KLNEEIAII	HLA-A*02:01, HLA-A*02:03	-0.01088
	471	EIAIILASF	HLA-A*34:01	**0.15254**
	478	SFSASTSAF	HLA-A*02:01, HLA-A*02:03, HLA-B*15:21	**0.01528**
	482	STSAFVETV	HLA-A*02:03	-0.08747
	483	TSAFVETVK	HLA-A*11:01	**0.07779**

	487	VETVKGLDY	HLA-B*18:01, HLA-B*44:03	5.00E-05
	488	ETVKGLDYK	HLA-A*34:01	-0.25068
	527	SILSPLYAF	HLA-B*15:02, HLA-B*15:21, HLA-B*35:05	-0.12032
	528	ILSPLYAFA	HLA-A*02:01, HLA-A*02:03	-0.04988
	533	YAFASEAAR	HLA-A*33:03	-0.02103
	541	RVVRSIFSR	HLA-A*11:01, HLA-A*33:03	**0.29466**
	552	ETAQNSVRV	HLA-A*34:01	-0.12893
	567	TILDGISQY	HLA-A*34:01, HLA-B*15:21	**0.12336**
	603	GVVQLTSQW	HLA-B*58:01	**0.09967**
	611	WLTNIFGTV	HLA-A*02:01, HLA-A*02:03	**0.10481**
	612	LTNIFGTVY	HLA-B*15:02, HLA-B*15:13, HLA-B*15:21	**0.13401**
	614	NIFGTVYEK	HLA-A*11:01, HLA-A*34:01	**0.08228**
	615	IFGTVYEKL	HLA-A*24:02, HLA-A*24:07	-0.09755
	618	TVYEKLKPV	HLA-A*34:01	-0.27795
	622	KLKPVLDWL	HLA-A*02:01, HLA-A*02:03	-0.0704
	640	FLRDGWEIV	HLA-A*02:03	**0.0312**
	669	EIKESVQTF	HLA-B*15:02, HLA-B*15:21	**0.00679**
	675	QTFFKLVNK	HLA-A*11:01, HLA-A*33:03	**0.03596**
	676	TFFKLVNKF	HLA-A*24:02, HLA-A*24:07	-0.24674
	679	KLVNKFLAL	HLA-A*02:01, HLA-A*02:03	-0.12198
	700	KALNLGETF	HLA-B*58:01	**0.26806**
	701	ALNLGETFV	HLA-A*02:01, HLA-A*02:03	**0.11952**
	708	FVTHSKGLY	HLA-A*34:01	**0.05558**
	709	VTHSKGLYR	HLA-A*33:03	**0.18902**
	724	EETGLLMPL	HLA-B*18:01, HLA-B*38:02, HLA-B*44:03	**0.2193**
	728	LLMPLKAPK	HLA-A*11:01	-0.22846
	743	GETLPTEVL	HLA-B*44:03	**0.01803**
	765	LEQPTSEAV	HLA-B*44:03	-0.19814
	790	LEIKDTEKY	HLA-B*18:01, HLA-B*44:03	**0.16486**
	804	NMMVTNNTF	HLA-B*15:02, HLA-B*15:13, HLA-B*15:21, HLA-B*35:05	**0.23433**
	806	MVTNNTFTL	HLA-A*34:01	**0.11013**
	807	VTNNTFTLK	HLA-A*11:01	**0.177**
	828	TVIEVQGYK	HLA-A*11:01, HLA-A*34:01	**0.33203**
	834	GYKSVNITF	HLA-A*24:02, HLA-A*24:07	-0.01928
	836	KSVNITFEL	HLA-B*58:01	**0.08112**
	847	RIDKVLNEK	HLA-A*11:01	**0.08112**
	851	VLNEKCSAY	HLA-B*15:02, HLA-B*15:21	-0.0521
	853	NEKCSAYTV	HLA-B*18:01	**0.29543**
	859	YTVELGTEV	HLA-A*34:01	-0.06319
	866	EVNEFACVV	HLA-A*34:01	**0.20722**

	877	AVIKTLQPV	HLA-A*02:01, HLA-A*02:03	**0.10025**
	880	KTLQPVSEL	HLA-B*58:01	-0.02077
	886	SELLTPLGI	HLA-B*44:03	-0.18359
	897	DEWSMATYY	HLA-B*18:01, HLA-B*44:03	-0.15173
	899	WSMATYYLF	HLA-A*24:02, HLA-A*24:07, HLA-B*15:02, HLA-B*15:13, HLA-B*15:21, HLA-B*18:01, HLA-B*35:05, HLA-B*58:01	**0.00742**
	905	YLFDESGEF	HLA-A*02:01, HLA-A*02:03, HLA-B*35:05	**0.14296**
	911	GEFKLASHM	HLA-B*18:01, HLA-B*44:03	-0.11053
	915	LASHMYCSF	HLA-B*15:02, HLA-B*15:13, HLA-B*15:21, HLA-B*35:05, HLA-B*58:01	-0.12588
	916	ASHMYCSFY	HLA-A*11:01	-0.1748
	938	EEFEPSTQY	HLA-B*44:03	-0.01086
	940	FEPSTQYEY	HLA-B*18:01, HLA-B*44:03	-0.29837
	946	YEYGTEDDY	HLA-B*18:01, HLA-B*44:03	**0.13906**
	953	DYQGKPLEF	HLA-A*24:02, HLA-A*24:07	**0.06337**
	959	LEFGATSAA	HLA-B*18:01	-0.17982
	999	TTIQTIVEV	HLA-A*34:01	-0.28092
	1005	VEVQPQLEM	HLA-B*18:01, HLA-B*44:03	**0.13404**
	1011	LEMELTPVV	HLA-B*18:01, HLA-B*38:02, HLA-B*44:03	-0.0627
	1022	IEVNSFSGY	HLA-B*18:01, HLA-B*44:03	-0.02539
	1023	EVNSFSGYL	HLA-A*34:01	-0.12922
	1030	YLKLTDNVY	HLA-B*15:02, HLA-B*15:21	-0.15837
	1032	KLTDNVYIK	HLA-A*11:01	**0.19528**
	1054	TVVVNAANV	HLA-A*34:01	**0.32162**
	1055	VVVNAANVY	HLA-B*15:02, HLA-B*15:21	**0.20214**
	1103	VLSGHNLAK	HLA-A*11:01	-0.16657
	1108	NLAKHCLHV	HLA-A*02:03	-0.00438
	1115	HVVGPNVNK	HLA-A*11:01	**0.12139**
	1134	YENFNQHEV	HLA-B*18:01, HLA-B*38:02, HLA-B*44:03	**0.05026**
	1140	HEVLLAPLL	HLA-B*18:01, HLA-B*38:02, HLA-B*44:03	**0.17137**
	1145	APLLSAGIF	HLA-B*35:05	**0.1294**
	1147	LLSAGIFGA	HLA-A*02:01	-0.18658
	1169	RTNVYLAVF	HLA-B*58:01	-0.20081
	1171	NVYLAVFDK	HLA-A*11:01	-0.32885
	1181	LYDKLVSSF	HLA-A*24:02, HLA-A*24:07	**0.02993**
	1184	KLVSSFLEM	HLA-B*15:02, HLA-B*15:21	-0.1653
	1185	LVSSFLEMK	HLA-A*11:01	**0.02089**
	1205	IPKEEVKPF	HLA-B*35:05	**0.08438**
	1213	FITESKPSV	HLA-A*02:03	-0.06827
	1216	ESKPSVEQR	HLA-A*33:03	**0.25781**

	1230	KIKACVEEV	HLA-A*02:03	-0.16448
	1254	LYIDINGNL	HLA-A*24:02, HLA-A*24:07	**0.2052**
	1269	LVSDIDITF	HLA-B*15:02, HLA-B*15:13, HLA-B*15:21, HLA-B*35:05, HLA-B*58:01	**0.2281**
	1277	FLKKDAPYI	HLA-A*02:03	-0.18386
	1295	LTAVVIPTK	HLA-A*11:01	**0.20624**
	1311	MLAKALRKV	HLA-A*02:03	**0.09181**
	1328	YPGQGLNGY	HLA-B*35:05	-0.04849
	1364	QEILGTVSW	HLA-B*18:01, HLA-B*44:03	-0.16397
	1366	ILGTVSWNL	HLA-A*02:01	**0.07671**
	1373	NLREMLAHA	HLA-A*02:03	**0.2227**
	1393	ETKAIVSTI	HLA-A*34:01	-0.45577
	1429	TVASLINTL	HLA-A*34:01	**0.33479**
	1432	SLINTLNDL	HLA-A*02:03	-0.4751
	1441	NETLVTMPL	HLA-B*18:01, HLA-B*38:02, HLA-B*44:03	-0.1606
	1498	EHFIETISL	HLA-B*38:02	-0.18593
	1502	ETISLAGSY	HLA-A*34:01	-0.02225
	1503	TISLAGSYK	HLA-A*11:01	-0.12476
	1520	TQLGIEFLK	HLA-A*11:01	-0.35677
	1526	FLKRGDKSV	HLA-A*02:03	**0.15015**
	1535	YYTSNPTTF	HLA-A*24:02, HLA-A*24:07	**0.3263**
	1537	TSNPTTFHL	HLA-B*58:01	**0.24962**
	1547	GEVITFDNL	HLA-B*44:03	**0.33519**
	1548	EVITFDNLK	HLA-A*34:01	-0.22258
	1554	NLKTLLSLR	HLA-A*33:03	-0.24009
	1562	REVRTIKVF	HLA-B*44:03	-0.2427
	1565	RTIKVFTTV	HLA-A*02:03, HLA-A*24:07, HLA-A*34:01	-0.27622
	1581	QVVDMSMTY	HLA-A*34:01, HLA-B*15:02, HLA-B*15:13, HLA-B*15:21, HLA-B*35:05	-0.1306
	1585	MSMTYGQQF	HLA-B*15:02, HLA-B*15:13, HLA-B*15:21, HLA-B*35:05, HLA-B*58:01	-0.24791
	1612	HEGKTFYVL	HLA-B*18:01, HLA-B*38:02, HLA-B*44:03	**0.25596**
	1625	TLRVEAFEY	HLA-B*15:02, HLA-B*15:21	-0.04828
	1633	YYHTTDPSF	HLA-A*24:02, HLA-A*24:07	**0.08212**
	1634	YHTTDPSFL	HLA-B*38:02	**0.44289**
	1641	FLGRYMSAL	HLA-A*02:01, HLA-A*02:03	**0.1259**
	1645	YMSALNHTK	HLA-A*11:01	**0.00668**
	1646	MSALNHTKK	HLA-A*11:01	-0.41789
	1647	SALNHTKKW	HLA-B*58:01	**0.16197**
	1674	YLATALLTL	HLA-A*02:01, HLA-A*02:03	**0.32837**

	1690	NPPALQDAY	HLA-B*35:05	**0.12163**
	1704	GEAANFCAL	HLA-B*38:02, HLA-B*44:03	**0.04611**
	1711	ALILAYCNK	HLA-A*11:01	**0.08979**
	1726	DVRETMSYL	HLA-A*34:01	-0.03442
	1732	SYLFQHANL	HLA-A*24:02, HLA-A*24:07	**0.28227**
	1765	EAVMYMGTL	HLA-A*34:01	**0.19398**
	1767	VMYMGTLSY	HLA-A*11:01, HLA-A*34:01, HLA-B*15:02, HLA-B*15:13, HLA-B*15:21, HLA-B*18:01, HLA-B*35:05, HLA-B*44:03, HLA-B*58:01	-0.03831
	1771	GTLSYEQFK	HLA-A*11:01	**0.24003**
	1772	TLSYEQFKK	HLA-A*11:01	-0.34814
	1786	CTCGKQATK	HLA-A*11:01	-0.08934
	1795	YLVQQESPF	HLA-B*15:02	**0.1401**
	1795	YLVQQESPF	HLA-B*15:21	-0.29683
	1805	MMSAPPAQY	HLA-B*15:02, HLA-B*15:13, HLA-B*15:21, HLA-B*35:05, HLA-B*58:01	**0.08593**
	1812	QYELKHGTF	HLA-A*24:02	-0.06759
	1818	GTFTCASEY	HLA-A*11:01, HLA-B*15:13, HLA-B*58:01	**0.32333**
	1822	CASEYTGNY	HLA-A*34:01, HLA-B*15:02, HLA-B*15:13, HLA-B*15:21, HLA-B*35:05	**0.18319**
	1837	HITSKETLY	HLA-A*34:01	**0.21142**
	1846	CIDGALLTK	HLA-A*11:01	-0.01178
	1864	DVFYKENSY	HLA-A*34:01	**0.14035**
	1868	KENSYTTTI	HLA-B*44:03	-0.10212
	1874	TTIKPVTYK	HLA-A*11:01, HLA-A*34:01	**0.15029**
	1898	YYKKDNSYF	HLA-A*24:02, HLA-A*24:07	**0.13333**
	1919	YPNASFDNF	HLA-B*15:13, HLA-B*35:05	-0.27369
	1928	KFVCDNIKF	HLA-A*24:02, HLA-A*24:07	**0.1358**
	1970	DYKHYTPSF	HLA-A*24:02	**0.31026**
	1983	KLLHKPIVW	HLA-B*58:01	-0.03961
	1999	KATYKPNTW	HLA-B*58:01	**0.02826**
	2001	TYKPNTWCI	HLA-A*24:02, HLA-A*24:07	**0.5572**
	2005	NTWCIRCLW	HLA-B*58:01	-0.47377
	2016	KPVETSNSF	HLA-B*15:02, HLA-B*15:13, HLA-B*15:21, HLA-B*35:05	**0.05262**
	2019	ETSNSFDVL	HLA-A*34:01	**0.17323**
	2020	TSNSFDVLK	HLA-A*11:01	**0.19998**
	2050	VVENPTIQK	HLA-A*11:01	-0.01973
	2068	TEVVGDIIL	HLA-B*18:01, HLA-B*38:02, HLA-B*44:03	-0.10878
	2075	ILKPANNSL	HLA-A*02:03	-0.11178
	2110	NELSRVLGL	HLA-B*18:01, HLA-B*38:02, HLA-B*44:03	**0.05689**
	2111	ELSRVLGLK	HLA-A*34:01	-0.19241

	2126	LAAVNSVPW	HLA-B*15:13, HLA-B*58:01	**0.09719**
	2132	VPWDTIANY	HLA-B*15:13, HLA-B*18:01, HLA-B*35:05	-0.08099
	2150	STTTNIVTR	HLA-A*33:03	-0.28472
	2162	RVCTNYMPY	HLA-A*11:01	-0.01179
	2164	CTNYMPYFF	HLA-B*58:01	-0.06736
	2166	NYMPYFFTL	HLA-A*24:02, HLA-A*24:07, HLA-B*38:02	**0.18574**
	2167	YMPYFFTLL	HLA-A*02:01, HLA-A*02:03, HLA-B*38:02	-0.23641
	2168	MPYFFTLLL	HLA-B*35:05, HLA-B*38:02	-0.037
	2175	LLQLCTFTR	HLA-A*33:03	**0.01243**
	2180	TFTRSTNSR	HLA-A*33:03	-0.10484
	2191	ASMPTTIAK	HLA-A*11:01	**0.00503**
	2195	TTIAKNTVK	HLA-A*11:01	**0.30249**
	2229	IIWFLLLSV	HLA-A*02:01	**0.15002**
	2241	SLIYSTAAL	HLA-A*02:03, HLA-B*15:21	-0.24334
	2253	MSNLGMPSY	HLA-A*11:01, HLA-A*34:01, HLA-B*15:02, HLA-B*15:13, HLA-B*15:21, HLA-B*35:05, HLA-B*58:01	**0.05143**
	2269	YLNSTNVTI	HLA-A*02:01, HLA-A*02:03	-0.16914
	2272	STNVTIATY	HLA-A*11:01, HLA-A*34:01, HLA-B*15:02, HLA-B*15:13, HLA-B*15:21, HLA-B*58:01	-0.00384
	2305	TIQITISSF	HLA-B*15:02, HLA-B*15:13, HLA-B*15:21	**0.0599**
	2307	QITISSFKW	HLA-B*58:01	**0.20408**
	2318	TAFGLVAEW	HLA-B*15:13, HLA-B*58:01	**0.01392**
	2322	LVAEWFLAY	HLA-A*11:01, HLA-A*34:01, HLA-B*15:02, HLA-B*15:13, HLA-B*15:21, HLA-B*18:01, HLA-B*35:05	-0.27126
	2324	AEWFLAYIL	HLA-B*18:01, HLA-B*38:02, HLA-B*44:03	**0.23318**
	2327	FLAYILFTR	HLA-A*33:03	-0.20225
	2328	LAYILFTRF	HLA-B*15:13, HLA-B*58:01	**0.11285**
	2329	AYILFTRFF	HLA-A*24:02, HLA-A*24:07	**0.28861**
	2331	ILFTRFFYV	HLA-A*02:01, HLA-A*02:03	-0.3399
	2332	LFTRFFYVL	HLA-A*24:02, HLA-A*24:07	-0.3573
	2334	TRFFYVLGL	HLA-A*11:01, HLA-B*15:02, HLA-B*15:21, HLA-B*38:02	**0.18696**
	2345	IMQLFFSYF	HLA-A*24:02, HLA-A*24:07, HLA-B*15:02	**0.06167**
	2347	QLFFSYFAV	HLA-A*02:01, HLA-A*02:03	**0.25374**
	2354	AVHFISNSW	HLA-B*58:01	-0.14475
	2356	HFISNSWLM	HLA-A*24:02, HLA-A*24:07, HLA-B*58:01	**0.03257**
	2357	FISNSWLMW	HLA-B*58:01	-0.10306
	2362	WLMWLIINL	HLA-A*02:01, HLA-A*02:03	-0.16383
	2363	LMWLIINLV	HLA-A*02:01	**0.02119**
	2371	VQMAPISAM	HLA-B*15:02, HLA-B*15:21	**0.04528**
	2372	QMAPISAMV	HLA-A*02:01, HLA-A*02:03	**0.14228**

	2381	RMYIFFASF	HLA-A*24:02, HLA-A*24:07, HLA-B*15:02, HLA-B*15:13, HLA-B*15:21, HLA-B*35:05, HLA-B*38:02, HLA-B*58:01	**0.08761**
	2382	MYIFFASFY	HLA-A*24:02	**0.06244**
	2383	YIFFASFYY	HLA-A*11:01, HLA-A*34:01, HLA-B*15:02, HLA-B*15:13, HLA-B*15:21, HLA-B*18:01, HLA-B*35:05	**0.3343**
	2384	IFFASFYYV	HLA-A*24:07, HLA-A*33:03	**0.11769**
	2385	FFASFYYVW	HLA-A*24:02, HLA-A*24:07	**0.08328**
	2391	YVWKSYVHV	HLA-A*02:01	-0.15127
	2405	SSTCMMCYK	HLA-A*11:01	-0.07566
	2406	STCMMCYKR	HLA-A*33:03	**0.12576**
	2408	CMMCYKRNR	HLA-A*33:03	**0.02228**
	2441	KGFCKLHNW	HLA-B*58:01	-0.14675
	2455	DTFCAGSTF	HLA-A*34:01, HLA-B*15:02, HLA-B*15:13, HLA-B*15:21	-0.11221
	2466	DEVARDLSL	HLA-B*18:01, HLA-B*38:02	**0.03366**
	2468	VARDLSLQF	HLA-B*15:02, HLA-B*15:13, HLA-B*35:05, HLA-B*58:01	-0.02137
	2487	SYIVDSVTV	HLA-A*24:07	-0.48075
	2488	YIVDSVTVK	HLA-A*34:01	**0.02392**
	2498	GSIHLYFDK	HLA-A*11:01	**0.0636**
	2512	YERHSLSHF	HLA-B*18:01, HLA-B*44:03	-0.13656
	2518	SHFVNLDNL	HLA-B*38:02	**0.19909**
	2519	HFVNLDNLR	HLA-A*33:03	-0.04273
	2551	SSAKSASVY	HLA-A*34:01, HLA-B*15:02, HLA-B*15:13, HLA-B*15:21	**0.11506**
	2552	SAKSASVYY	HLA-B*15:02, HLA-B*15:13, HLA-B*15:21	-0.0981
	2568	ILLLDQALV	HLA-A*02:01	-0.07971
	2589	MFDAYVNTF	HLA-A*24:02, HLA-A*24:07, HLA-B*35:05	**0.11817**
	2593	YVNTFSSTF	HLA-A*34:01, HLA-B*15:02, HLA-B*15:13, HLA-B*15:21, HLA-B*35:05	**0.22019**
	2595	NTFSSTFNV	HLA-A*34:01	-0.19434
	2597	FSSTFNVPM	HLA-B*15:21, HLA-B*35:05	**0.08367**
	2599	STFNVPMEK	HLA-A*11:01, HLA-A*33:03, HLA-A*34:01	**0.09901**
	2607	KLKTLVATA	HLA-A*02:03	-0.08019
	2613	ATAEAELAK	HLA-A*11:01	**0.0548**
	2617	AELAKNVSL	HLA-B*44:03	-0.03902
	2628	VLSTFISAA	HLA-A*02:03	**0.29793**
	2629	LSTFISAAR	HLA-A*33:03, HLA-A*34:01	-0.21317
	2646	ETKDVVECL	HLA-A*34:01	-0.08793
	2671	NYMLTYNKV	HLA-A*24:02, HLA-A*24:07	**0.1614**
	2704	KSHNIALIW	HLA-B*58:01	**0.09873**
	2718	MSLSEQLRK	HLA-A*11:01	-0.2627
	2748	QVVNVVTTK	HLA-A*11:01, HLA-A*34:01	**0.236**

	2771	QLIKVTLVF	HLA-B*15:02, HLA-B*15:13, HLA-B*15:21	**0.08004**
	2778	VFLFVAAIF	HLA-A*24:02, HLA-A*24:07	**0.26401**
	2779	FLFVAAIFY	HLA-B*15:02, HLA-B*15:21	-0.21168
	2781	FVAAIFYLI	HLA-A*02:01	**0.09674**
	2784	AIFYLITPV	HLA-A*02:01, HLA-A*02:03, HLA-A*34:01	**0.05282**
	2786	FYLITPVHV	HLA-A*24:02, HLA-A*24:07	-0.05505
	2787	YLITPVHVM	HLA-A*02:01, HLA-A*02:03, HLA-B*15:02, HLA-B*15:21	**0.28124**
	2789	ITPVHVMSK	HLA-A*11:01	**0.43221**
	2793	HVMSKHTDF	HLA-B*15:02, HLA-B*15:21	-0.07818
	2797	KHTDFSSEI	HLA-B*38:02	**0.1586**
	2801	FSSEIIGYK	HLA-A*11:01, HLA-A*34:01	**0.15656**
	2821	STDTCFANK	HLA-A*11:01	**0.14212**
	2832	DFDTWFSQR	HLA-A*33:03	-0.13194
	2875	TTNGDFLHF	HLA-B*15:13, HLA-B*58:01	-0.07941
	2883	FLPRVFSAV	HLA-A*02:01, HLA-A*02:03	-0.15483
	2888	FSAVGNICY	HLA-B*15:02, HLA-B*15:13, HLA-B*15:21	-0.08512
	2896	YTPSKLIEY	HLA-A*34:01	-0.13343
	2900	KLIEYTDFA	HLA-A*02:01	-0.11782
	2914	LAAECTIFK	HLA-A*11:01	-0.28981
	2936	NVLEGSVAY	HLA-A*34:01, HLA-B*15:02, HLA-B*15:21, HLA-B*18:01, HLA-B*35:05	-0.1059
	2946	SLRPDTRYV	HLA-A*02:03	**0.0827**
	2959	SIIQFPNTY	HLA-A*34:01, HLA-B*15:02, HLA-B*15:21	**0.24538**
	2970	GSVRVVTTF	HLA-B*15:13, HLA-B*58:01	**0.22152**
	2976	TTFDSEYCR	HLA-A*11:01, HLA-A*33:03, HLA-A*34:01	**0.15864**
	3003	WVLNNDYYR	HLA-A*33:03	-0.21208
	3009	YYRSLPGVF	HLA-A*24:02, HLA-A*24:07	**0.23882**
	3012	SLPGVFCGV	HLA-A*02:01, HLA-A*02:03	**0.33033**
	3015	GVFCGVDAV	HLA-A*02:03	**0.00599**
	3025	LLTNMFTPL	HLA-A*02:01, HLA-A*02:03	**0.00758**
	3029	MFTPLIQPI	HLA-A*24:02, HLA-A*24:07	**0.0579**
	3053	AIVVTCLAY	HLA-B*15:02, HLA-B*15:21	-0.17322
	3059	LAYYFMRFR	HLA-A*33:03	-0.30428
	3060	AYYFMRFRR	HLA-A*33:03	-0.0587
	3071	GEYSHVVAF	HLA-B*15:02, HLA-B*18:01, HLA-B*38:02, HLA-B*44:03	-0.12439
	3074	SHVVAFNTL	HLA-B*38:02	**0.24313**
	3075	HVVAFNTLL	HLA-A*34:01	-0.26543
	3076	VVAFNTLLF	HLA-A*24:07	**0.27821**
	3082	LLFLMSFTV	HLA-A*02:01, HLA-A*02:03	-0.07943
	3085	LMSFTVLCL	HLA-A*02:01	**0.13402**

	3088	FTVLCLTPV	HLA-A*02:01, HLA-A*02:03, HLA-A*34:01	-0.34574
	3089	TVLCLTPVY	HLA-B*15:21, HLA-B*35:05	-0.13812
	3099	FLPGVYSVI	HLA-A*02:01, HLA-A*02:03	-0.33779
	3100	LPGVYSVIY	HLA-B*35:05	**0.29512**
	3102	GVYSVIYLY	HLA-A*11:01, HLA-A*34:01, HLA-B*58:01	**0.05586**
	3103	VYSVIYLYL	HLA-A*24:02, HLA-A*24:07	**0.02998**
	3105	SVIYLYLTF	HLA-A*24:02, HLA-A*24:07, HLA-B*15:02, HLA-B*15:13	**0.13713**
	3107	IYLYLTFYL	HLA-A*24:02, HLA-A*24:07	-0.15716
	3113	FYLTNDVSF	HLA-A*24:02, HLA-A*24:07	-0.21463
	3114	YLTNDVSFL	HLA-A*02:01, HLA-A*02:03	-0.26262
	3119	VSFLAHIQW	HLA-B*58:01	**0.08425**
	3121	FLAHIQWMV	HLA-A*02:01, HLA-A*02:03	**0.2064**
	3129	VMFTPLVPF	HLA-B*15:02, HLA-B*15:13, HLA-B*15:21	**0.10163**
	3135	VPFWITIAY	HLA-B*15:02, HLA-B*15:13, HLA-B*15:21, HLA-B*18:01, HLA-B*35:05	-0.05933
	3137	FWITIAYII	HLA-A*24:02, HLA-A*24:07	**0.08626**
	3146	CISTKHFYW	HLA-B*58:01	**0.30988**
	3147	ISTKHFYWF	HLA-A*24:02, HLA-A*24:07, HLA-B*58:01	**0.14443**
	3148	STKHFYWFF	HLA-A*24:02, HLA-A*24:07	**0.00629**
	3151	HFYWFFSNY	HLA-A*33:03, HLA-A*34:01, HLA-B*18:01	-0.18993
	3152	FYWFFSNYL	HLA-A*24:02, HLA-A*24:07	-0.05002
	3153	YWFFSNYLK	HLA-A*11:01, HLA-A*33:03	-0.0549
	3154	WFFSNYLKR	HLA-A*33:03	**0.34464**
	3158	NYLKRRVVF	HLA-A*24:02, HLA-A*24:07	**0.2888**
	3163	RVVFNGVSF	HLA-B*15:02, HLA-B*15:21	-0.17841
	3174	FEEAALCTF	HLA-B*18:01, HLA-B*44:03	-0.01434
	3178	ALCTFLLNK	HLA-A*11:01	**0.24386**
	3182	FLLNKEMYL	HLA-A*02:01, HLA-A*02:03	**0.09072**
	3183	LLNKEMYLK	HLA-A*11:01	-0.25542
	3198	LPLTQYNRY	HLA-B*35:05	-0.2965
	3201	TQYNRYLAL	HLA-B*38:02	**0.05676**
	3205	RYLALYNKY	HLA-A*24:02, HLA-A*24:07	**0.26255**
	3207	LALYNKYKY	HLA-B*35:05	-0.02843
	3218	GAMDTTSYR	HLA-A*11:01, HLA-A*33:03, HLA-A*34:01	**0.24273**
	3225	YREAACCHL	HLA-B*38:02	**0.07994**
	3232	HLAKALNDF	HLA-B*15:02, HLA-B*15:21	**0.0999**
	3248	LYQPPQTSI	HLA-A*24:02, HLA-A*24:07	**0.30368**
	3259	AVLQSGFRK	HLA-A*11:01	**0.00581**
	3267	KMAFPSGKV	HLA-A*02:03	-0.05604
	3285	GTTTLNGLW	HLA-B*58:01	**0.06748**

	3308	SEDMLNPNY	HLA-B*18:01, HLA-B*44:03	**0.24122**
	3311	MLNPNYEDL	HLA-A*02:03	**0.19857**
	3342	HSMQNCVLK	HLA-A*11:01	**0.10269**
	3359	KTPKYKFVR	HLA-A*33:03	**0.04133**
	3372	QTFSVLACY	HLA-A*11:01, HLA-A*34:01, HLA-B*15:02, HLA-B*15:13, HLA-B*15:21, HLA-B*58:01	-0.06765
	3390	CAMRPNFTI	HLA-B*58:01	**0.19601**
	3402	FLNGSCGSV	HLA-A*02:01, HLA-A*02:03	-0.23637
	3419	VSFCYMHHM	HLA-B*58:01	**0.10982**
	3439	LEGNFYGPF	HLA-B*18:01, HLA-B*44:03	-0.23124
	3442	NFYGPFVDR	HLA-A*33:03	-0.07718
	3462	ITVNVLAWL	HLA-B*58:01	**0.16015**
	3463	TVNVLAWLY	HLA-A*11:01	**0.25621**
	3466	VLAWLYAAV	HLA-A*02:01, HLA-A*02:03	**0.23304**
	3472	AAVINGDRW	HLA-B*58:01	**0.05863**
	3481	FLNRFTTTL	HLA-A*02:01, HLA-A*02:03	-0.05892
	3507	DHVDILGPL	HLA-B*38:02	**0.3346**
	3523	VLDMCASLK	HLA-A*11:01	-0.32096
	3541	RTILGSALL	HLA-B*58:01	**0.45285**
	3548	LLEDEFTPF	HLA-B*15:02, HLA-B*15:21	**0.10642**
	3551	DEFTPFDVV	HLA-B*18:01	-0.14748
	3552	EFTPFDVVR	HLA-A*33:03	-0.26551
	3564	GVTFQSAVK	HLA-A*11:01	**0.2541**
	3565	VTFQSAVKR	HLA-A*33:03	-0.13471
	3573	RTIKGTHHW	HLA-A*24:07, HLA-B*15:13, HLA-B*58:01	**0.11065**
	3578	THHWLLLTI	HLA-B*38:02	-0.41802
	3579	HHWLLLTIL	HLA-B*38:02	**0.15845**
	3582	LLLTILTSL	HLA-A*02:01, HLA-A*02:03	**0.21385**
	3586	ILTSLLVLV	HLA-A*02:01, HLA-A*02:03	-0.05472
	3591	LVLVQSTQW	HLA-B*58:01	-0.0808
	3595	QSTQWSLFF	HLA-B*58:01	-0.2325
	3596	STQWSLFFF	HLA-A*24:07	-0.02691
	3597	TQWSLFFFL	HLA-A*02:01, HLA-B*38:02	**0.0593**
	3605	LYENAFLPF	HLA-A*24:02, HLA-A*24:07	-0.27148
	3618	IAMSAFAMM	HLA-B*35:05	**0.02816**
	3620	MSAFAMMFV	HLA-A*34:01	**0.09905**
	3621	SAFAMMFVK	HLA-A*11:01	**0.01168**
	3638	FLLPSLATV	HLA-A*02:01, HLA-A*02:03	-0.08907
	3640	LPSLATVAY	HLA-B*15:02, HLA-B*15:21, HLA-B*18:01, HLA-B*35:05	**0.35609**
	3645	TVAYFNMVY	HLA-A*11:01, HLA-A*34:01, HLA-B*15:02, HLA-B*15:21,	-0.01723

			HLA-B*35:05	
	3652	VYMPASWVM	HLA-A*24:02, HLA-A*24:07	-0.25896
	3654	MPASWVMRI	HLA-B*35:05	-0.39659
	3657	SWVMRIMTW	HLA-A*24:02, HLA-A*24:07	-0.1867
	3661	RIMTWLDMV	HLA-A*02:01, HLA-A*02:03	-0.12088
	3671	TSLSGFKLK	HLA-A*11:01	**0.07442**
	3677	KLKDCVMYA	HLA-A*02:03	-0.1898
	3683	MYASAVVLL	HLA-A*24:02, HLA-A*24:07	**0.07782**
	3684	YASAVVLLI	HLA-B*58:01	-0.12397
	3692	ILMTARTVY	HLA-B*15:02, HLA-B*15:21, HLA-B*35:05	-0.05113
	3709	TLMNVLTLV	HLA-A*02:01, HLA-A*02:03	-0.07023
	3710	LMNVLTLVY	HLA-B*15:02, HLA-B*15:13, HLA-B*15:21, HLA-B*35:05 HLA-B*58:01	-0.01717
	3731	SMWALIISV	HLA-A*02:01, HLA-A*02:03, HLA-B*38:02	-0.43574
	3735	LIISVTSNY	HLA-A*34:01, HLA-B*15:02, HLA-B*15:13, HLA-B*15:21	**0.2513**
	3752	FLARGIVFM	HLA-A*02:01, HLA-A*02:03	-0.13454
	3762	VEYCPIFFI	HLA-B*44:03	-0.0765
	3774	TLQCIMLVY	HLA-B*15:21	-0.06755
	3779	MLVYCFLGY	HLA-A*34:01, HLA-B*15:02, HLA-B*15:21, HLA-B*35:05	-0.16192
	3791	CYFGLFCLL	HLA-A*24:02, HLA-A*24:07	-0.16073
	3811	DYLVSTQEF	HLA-A*24:02	-0.15508
	3819	FRYMNSQGL	HLA-B*38:02	-0.25158
	3838	KLNIKLLGV	HLA-A*02:01, HLA-A*02:03	-0.38114
	3870	VLLSVLQQL	HLA-A*02:01	**0.007**
	3879	RVESSSKLW	HLA-B*58:01	-0.4958
	3885	KLWAQCVQL	HLA-A*02:01, HLA-A*02:03	**0.1065**
	3898	LLAKDTTEA	HLA-A*02:03	-0.19245
	3899	LAKDTTEAF	HLA-B*15:02, HLA-B*15:13, HLA-B*15:21, HLA-B*35:05	-0.02118
	3905	EAFEKMVSL	HLA-A*34:01	**0.15781**
	3909	KMVSLLSVL	HLA-A*02:01, HLA-A*02:03	-0.04845
	3912	SLLSVLLSM	HLA-A*02:01	**0.24061**
	3945	SEFSSLPSY	HLA-B*15:02, HLA-B*15:13, HLA-B*15:21, HLA-B*18:01, HLA-B*44:03	**0.12602**
	3948	SSLPSYAAF	HLA-A*24:02, HLA-A*24:07, HLA-B*15:02, HLA-B*15:13, HLA-B*15:21, HLA-B*35:05, HLA-B*58:01	-0.22896
	3982	SLNVAKSEF	HLA-B*15:02, HLA-B*15:21	-0.07037
	3988	SEFDRDAAM	HLA-B*18:01, HLA-B*38:02, HLA-B*44:03	-0.12288
	4000	LEKMADQAM	HLA-B*18:01	-0.0765
	4003	MADQAMTQM	HLA-B*35:05	-0.07746
	4008	MTQMYKQAR	HLA-A*33:03	**0.1801**

	4027	AMQTMLFTM	HLA-A*02:01	**0.20414**
	4028	MQTMLFTML	HLA-B*38:02	-0.21401
	4029	QTMLFTMLR	HLA-A*11:01, HLA-A*33:03<br>HLA-A*34:01	-0.37437
	4030	TMLFTMLRK	HLA-A*11:01	**0.02386**
	4031	MLFTMLRKL	HLA-A*02:01, HLA-A*02:03	-0.19828
	4043	ALNNIINNA	HLA-A*02:03	-0.23625
	4071	VVIPDYNTY	HLA-B*15:02, HLA-B*15:13, HLA-B*15:21, HLA-B*35:05	-0.01197
	4072	VIPDYNTYK	HLA-A*11:01	**0.16112**
	4086	TTFTYASAL	HLA-A*34:01	**0.03347**
	4087	TFTYASALW	HLA-A*24:02, HLA-A*24:07	**0.14013**
	4089	TYASALWEI	HLA-A*24:02, HLA-A*24:07	**0.06014**
	4093	ALWEIQQVV	HLA-A*02:01, HLA-A*02:03	-0.04249
	4138	LQNNELSPV	HLA-A*02:03	-0.2515
	4167	ALAYYNTTK	HLA-A*11:01	-0.15801
	4182	ALLSDLQDL	HLA-A*02:01	**0.25478**
	4184	LSDLQDLKW	HLA-B*58:01	**0.14399**
	4186	DLQDLKWAR	HLA-A*33:03	**0.19412**
	4189	DLKWARFPK	HLA-A*33:03	**0.1602**
	4197	KSDGTGTIY	HLA-B*58:01	**0.03212**
	4206	TELEPPCRF	HLA-B*18:01, HLA-B*44:03	**0.17636**
	4228	YFIKGLNNL	HLA-A*24:02, HLA-A*24:07	-0.06252
	4260	PANSTVLSF	HLA-B*15:13	-0.1653
	4263	STVLSFCAF	HLA-B*15:02, HLA-B*15:13, HLA-B*15:21, HLA-B*35:05,<br>HLA-B*58:01	**0.15992**
	4265	VLSFCAFAV	HLA-A*02:01	-0.19793
	4271	FAVDAAKAY	HLA-A*34:01, HLA-B*15:02, HLA-B*15:13, HLA-B*15:21,<br>HLA-B*18:01, HLA-B*35:05	**0.03414**
	4272	AVDAAKAYK	HLA-A*11:01	-0.34838
	4282	YLASGGQPI	HLA-A*02:01, HLA-A*02:03	**0.03976**
	4363	TLKNTVCTV	HLA-A*02:03	**0.21633**
	4401	RVCGVSAAR	HLA-A*33:03	**0.28943**
	4424	RAFDIYNDK	HLA-A*11:01	-0.12238
	4428	IYNDKVAGF	HLA-A*24:02, HLA-A*24:07	**0.30199**
	4455	NLIDSYFVV	HLA-A*02:01, HLA-A*02:03	-0.19751
	4456	LIDSYFVVK	HLA-A*11:01	**0.11288**
	4460	YFVVKRHTF	HLA-A*24:02, HLA-A*24:07	-0.0823
	4472	QHEETIYNL	HLA-B*38:02	-0.19232
	4473	HEETIYNLL	HLA-B*18:01, HLA-B*38:02	-0.09417
	4479	NLLKDCPAV	HLA-A*02:01	-0.26518

	4484	CPAVAKHDF	HLA-B*35:05	**0.28136**
	4486	AVAKHDFFK	HLA-A*11:01	**0.01505**
	4487	VAKHDFFKF	HLA-A*24:02, HLA-A*24:07, HLA-B*15:13	-0.03358
	4501	MVPHISRQR	HLA-A*33:03	-0.09719
	4513	YTMADLVYA	HLA-A*02:01	-0.0723
	4514	TMADLVYAL	HLA-A*02:01	-0.41653
	4514	TMADLVYAL	HLA-A*02:03, HLA-A*34:01, HLA-B*38:02	**0.14139**
	4515	MADLVYALR	HLA-A*33:03	-0.10693
	4532	TLKEILVTY	HLA-B*15:02, HLA-B*15:21	**0.19394**
	4557	VENPDILRV	HLA-B*44:03	-0.21888
	4580	TVQFCDAMR	HLA-A*33:03	**0.37218**
	4627	SYYSLLMPI	HLA-A*24:02, HLA-A*24:07	**0.10786**
	4628	YYSLLMPIL	HLA-A*24:02	**0.17082**
	4644	AESHVDTDL	HLA-B*44:03	-0.0358
	4656	YIKWDLLKY	HLA-B*15:02, HLA-B*15:21	**0.07068**
	4672	KLFDRYFKY	HLA-A*11:01, HLA-B*15:02, HLA-B*15:21	**0.01651**
	4677	YFKYWDQTY	HLA-B*15:02	-0.127
	4685	YHPNCVNCL	HLA-B*38:02	**0.10344**
	4698	ILHCANFNV	HLA-A*02:01, HLA-A*02:03	**0.01828**
	4699	LHCANFNVL	HLA-B*38:02	-0.36257
	4712	FPPTSFGPL	HLA-B*35:05	-0.10238
	4715	TSFGPLVRK	HLA-A*11:01	-0.38391
	4723	KIFVDGVPF	HLA-B*15:02	**0.29328**
	4725	FVDGVPFVV	HLA-A*02:01	-0.26109
	4729	VPFVVSTGY	HLA-B*15:02, HLA-B*15:21, HLA-B*35:05	**0.23144**
	4731	FVVSTGYHF	HLA-B*15:02, HLA-B*15:13, HLA-B*15:21, HLA-B*35:05	-0.05806
	4732	VVSTGYHFR	HLA-A*11:01, HLA-A*33:03	**0.03204**
	4751	NLHSSRLSF	HLA-B*15:02, HLA-B*15:21	-0.06415
	4757	LSFKELLVY	HLA-B*15:02, HLA-B*15:13, HLA-B*15:21, HLA-B*35:05, HLA-B*58:01	**0.08254**
	4763	LVYAADPAM	HLA-B*15:02, HLA-B*15:21, HLA-B*35:05	-0.04305
	4771	MHAASGNLL	HLA-B*38:02	-0.1429
	4788	SVAALTNNV	HLA-A*34:01	-0.63928
	4790	AALTNNVAF	HLA-B*35:05	**0.09498**
	4800	TVKPGNFNK	HLA-A*11:01, HLA-A*33:03	**0.0318**
	4805	NFNKDFYDF	HLA-A*24:02	-0.10997
	4809	DFYDFAVSK	HLA-A*33:03	-0.13601
	4813	FAVSKGFFK	HLA-A*11:01	**0.09674**
	4840	AISDYDYYR	HLA-A*11:01, HLA-A*33:03	-0.32142
	4846	YYRYNLPTM	HLA-A*24:02	-0.25007

	4858	RQLLFVVEV	HLA-A*02:01	-0.19953
	4862	FVVEVVDKY	HLA-A*34:01, HLA-B*15:21	**0.18748**
	4891	KSAGFPFNK	HLA-A*11:01	-0.40603
	4892	SAGFPFNKW	HLA-B*58:01	**0.02304**
	4904	RLYYDSMSY	HLA-A*11:01, HLA-B*15:02, HLA-B*15:13, HLA-B*15:21, HLA-B*35:05	**0.06159**
	4913	EDQDALFAY	HLA-B*18:01	-0.25565
	4916	DALFAYTKR	HLA-A*33:03	**0.25237**
	4925	NVIPTITQM	HLA-A*34:01	**0.07774**
	4952	SICSTMTNR	HLA-A*33:03	**0.22046**
	4957	MTNRQFHQK	HLA-A*11:01, HLA-A*33:03	-0.28249
	4960	RQFHQKLLK	HLA-A*11:01	-0.11496
	4966	LLKSIAATR	HLA-A*33:03	**0.04742**
	4976	ATVVIGTSK	HLA-A*11:01	-0.17157
	4977	TVVIGTSKF	HLA-B*15:13	**0.16115**
	4981	GTSKFYGGW	HLA-B*58:01	**0.19545**
	4984	KFYGGWHNM	HLA-A*24:02, HLA-A*24:07	**0.07155**
	4985	FYGGWHNML	HLA-A*24:02, HLA-A*24:07	-0.0292
	4992	MLKTVYSDV	HLA-A*02:03	-0.24633
	5000	VENPHLMGW	HLA-B*44:03	-0.20658
	5002	NPHLMGWDY	HLA-B*35:05	**0.1552**
	5004	HLMGWDYPK	HLA-A*11:01, HLA-A*33:03, HLA-A*34:01	**0.1053**
	5015	RAMPNMLRI	HLA-A*24:07, HLA-B*58:01	**0.19848**
	5021	LRIMASLVL	HLA-B*38:02	-0.1188
	5023	IMASLVLAR	HLA-A*33:03	-0.26388
	5024	MASLVLARK	HLA-A*11:01, HLA-A*33:03, HLA-A*34:01	**0.24141**
	5034	TTCCSLSHR	HLA-A*33:03	**0.07388**
	5037	CSLSHRFYR	HLA-A*11:01, HLA-A*33:03	-0.44407
	5045	RLANECAQV	HLA-A*02:01, HLA-A*02:03	**0.16358**
	5057	MVMCGGSLY	HLA-A*11:01, HLA-A*34:01, HLA-B*15:02, HLA-B*15:13, HLA-B*15:21, HLA-B*35:05	-0.12346
	5058	VMCGGSLYV	HLA-A*02:01, HLA-A*02:03	**0.00065**
	5072	SSGDATTAY	HLA-B*15:02, HLA-B*15:21	-0.42561
	5077	TTAYANSVF	HLA-B*15:13, HLA-B*15:21	-0.54507
	5079	AYANSVFNI	HLA-A*24:02, HLA-A*24:07	**0.1332**
	5083	SVFNICQAV	HLA-A*02:03, HLA-A*34:01	-0.02845
	5131	DFVNEFYAY	HLA-B*18:01	**0.32841**
	5132	FVNEFYAYL	HLA-A*02:01, HLA-A*02:03, HLA-A*34:01	**0.25822**
	5138	AYLRKHFSM	HLA-A*24:02, HLA-A*24:07	**0.18095**
	5139	YLRKHFSMM	HLA-A*02:03, HLA-A*34:01, HLA-B*15:02, HLA-B*15:21	**0.24571**

	5149	LSDDAVVCF	HLA-B*58:01	**0.22816**
	5153	AVVCFNSTY	HLA-A*34:01, HLA-B*15:02<br>HLA-B*15:21	-0.0713
	5176	VLYYQNNVF	HLA-B*15:02, HLA-B*15:21	**0.06857**
	5177	LYYQNNVFM	HLA-A*24:02, HLA-A*24:07	**0.03544**
	5201	HEFCSQHTM	HLA-B*18:01, HLA-B*38:02, HLA-B*44:03	**0.03852**
	5245	LMIERFVSL	HLA-A*02:01, HLA-A*02:03, HLA-B*15:02, HLA-B*15:21, HLA-B*38:02	-0.11314
	5247	IERFVSLAI	HLA-B*44:03	-0.21713
	5250	FVSLAIDAY	HLA-A*34:01, HLA-B*15:02, HLA-B*15:21, HLA-B*35:05	**0.00813**
	5252	SLAIDAYPL	HLA-A*02:01	**0.06109**
	5254	AIDAYPLTK	HLA-A*11:01	-0.34359
	5266	QEYADVFHL	HLA-B*18:01, HLA-B*38:02, HLA-B*44:03	**0.19168**
	5267	EYADVFHLY	HLA-A*24:02, HLA-A*24:07, HLA-A*33:03, HLA-A*34:01, HLA-B*18:01	**0.00446**
	5270	DVFHLYLQY	HLA-A*34:01, HLA-B*15:21	-0.12346
	5271	VFHLYLQYI	HLA-A*24:07	**0.09962**
	5273	HLYLQYIRK	HLA-A*11:01	**0.06065**
	5274	LYLQYIRKL	HLA-A*24:02, HLA-A*24:07	**0.2989**
	5289	HMLDMYSVM	HLA-B*15:02, HLA-B*15:21	-0.2824
	5290	MLDMYSVML	HLA-A*02:01	-0.162
	5298	LTNDNTSRY	HLA-A*34:01	-0.08221
	5307	WEPEFYEAM	HLA-B*18:01	**0.22547**
	5308	EPEFYEAMY	HLA-B*35:05	**0.17402**
	5322	LQAVGACVL	HLA-B*38:02	-0.0303
	5356	HVISTSHKL	HLA-A*34:01	**0.02312**
	5363	KLVLSVNPY	HLA-B*15:02, HLA-B*15:21	-0.13664
	5364	LVLSVNPYV	HLA-A*02:01	-0.0681
	5379	DVTDVTQLY	HLA-A*34:01	**0.10385**
	5385	QLYLGGMSY	HLA-B*15:02, HLA-B*15:21	**0.20743**
	5391	MSYYCKSHK	HLA-A*11:01	-0.02267
	5396	KSHKPPISF	HLA-B*58:01	-0.00747
	5398	HKPPISFPL	HLA-B*38:02	-0.11279
	5454	KLFAAETLK	HLA-A*11:01	**0.34997**
	5462	KATEETFKL	HLA-B*58:01	-0.09616
	5464	TEETFKLSY	HLA-B*18:01, HLA-B*44:03	**0.00883**
	5466	ETFKLSYGI	HLA-A*34:01	**0.08282**
	5469	KLSYGIATV	HLA-A*02:01, HLA-A*02:03	**0.08282**
	5470	LSYGIATVR	HLA-A*33:03	**0.00758**
	5476	TVREVLSDR	HLA-A*33:03	-0.16766

	5478	REVLSDREL	HLA-B*44:03	**0.34667**
	5484	RELHLSWEV	HLA-B*18:01, HLA-B*38:02, HLA-B*44:03	**0.17203**
	5515	KVQIGEYTF	HLA-A*24:02, HLA-A*24:07, HLA-B*58:01	**0.05271**
	5517	QIGEYTFEK	HLA-A*11:01	**0.2015**
	5526	GDYGDAVVY	HLA-B*44:03	**0.32183**
	5527	DYGDAVVYR	HLA-A*33:03	**0.11594**
	5532	VVYRGTTTY	HLA-A*11:01, HLA-A*34:01<br>HLA-B*15:02, HLA-B*15:13, HLA-B*15:21, HLA-B*35:05, HLA-B*58:01	-0.21535
	5534	YRGTTTYKL	HLA-B*38:02	**0.17878**
	5541	KLNVGDYFV	HLA-A*02:01, HLA-A*02:03	-0.00533
	5548	FVLTSHTVM	HLA-B*15:02, HLA-B*15:21, HLA-B*35:05	-0.07451
	5562	TLVPQEHYV	HLA-A*02:01, HLA-A*02:03	-0.36401
	5568	HYVRITGLY	HLA-A*34:01	-0.04115
	5584	EFSSNVANY	HLA-A*34:01	-0.00647
	5586	SSNVANYQK	HLA-A*11:01	-0.07664
	5592	YQKVGMQKY	HLA-B*15:02, HLA-B*15:13, HLA-B*15:21	-0.11514
	5612	SHFAIGLAL	HLA-B*38:02	**0.18306**
	5614	FAIGLALYY	HLA-B*15:13, HLA-B*15:21, HLA-B*35:05, HLA-B*58:01	**0.07705**
	5618	LALYYPSAR	HLA-A*33:03	**0.17334**
	5619	ALYYPSARI	HLA-A*02:03	-0.02207
	5629	YTACSHAAV	HLA-A*02:03, HLA-A*34:01	-0.00719
	5635	AAVDALCEK	HLA-A*11:01	**0.10155**
	5670	KVNSTLEQY	HLA-B*58:01	**0.04077**
	5675	LEQYVFCTV	HLA-B*18:01, HLA-B*44:03	-0.01735
	5678	YVFCTVNAL	HLA-A*02:01, HLA-A*02:03, HLA-A*34:01, HLA-B*15:02, HLA-B*15:21, HLA-B*35:05, HLA-B*38:02	**0.24593**
	5688	ETTADIVVF	HLA-A*34:01	-0.021
	5697	DEISMATNY	HLA-B*18:01, HLA-B*44:03	**0.02065**
	5699	ISMATNYDL	HLA-B*58:01	-0.04876
	5713	RLRAKHYVY	HLA-B*15:02, HLA-B*15:21	**0.11471**
	5720	VYIGDPAQL	HLA-A*24:02, HLA-A*24:07	-0.30937
	5740	LEPEYFNSV	HLA-B*18:01	**0.13524**
	5753	KTIGPDMFL	HLA-B*58:01	**0.11933**
	5758	DMFLGTCRR	HLA-A*33:03	**0.10452**
	5758	DMFLGTCRR	HLA-A*34:01	**0.24348**
	5770	EIVDTVSAL	HLA-A*34:01	**0.06715**
	5771	IVDTVSALV	HLA-A*02:01	-0.21402
	5792	AQCFKMFYK	HLA-A*11:01	-0.20445
	5826	NPAWRKAVF	HLA-B*35:05	-0.1361

	5923	LQAENVTGL	HLA-A*02:01, HLA-A*02:03, HLA-B*38:02	**0.1545**
	5930	GLFKDCSKV	HLA-A*02:01, HLA-A*02:03	-0.1206
	5963	CVDIPGIPK	HLA-A*11:01	-0.12036
	5973	MTYRRLISM	HLA-A*34:01	-0.17386
	5977	RLISMMGFK	HLA-A*11:01	**0.22623**
	5980	SMMGFKMNY	HLA-A*11:01, HLA-A*34:01, HLA-B*15:02, HLA-B*15:13, HLA-B*15:21, HLA-B*35:05	-0.11462
	5988	YQVNGYPNM	HLA-B*15:02, HLA-B*15:21, HLA-B*38:02	**0.30201**
	6014	DVEGCHATR	HLA-A*33:03	**0.30187**
	6023	EAVGTNLPL	HLA-A*34:01	**0.03963**
	6031	LQLGFSTGV	HLA-A*02:01, HLA-A*02:03	**0.27719**
	6041	LVAVPTGYV	HLA-A*34:01	-0.33127
	6068	DQFKHLIPL	HLA-B*38:02	-0.30575
	6077	MYKGLPWNV	HLA-A*24:02	-0.27886
	6093	MLSDTLKNL	HLA-A*02:01, HLA-A*02:03	**0.17009**
	6100	NLSDRVVFV	HLA-A*02:01, HLA-A*02:03	**0.01931**
	6106	VFVLWAHGF	HLA-A*24:02, HLA-A*24:07	**0.10788**
	6108	VLWAHGFEL	HLA-A*02:01	**0.33197**
	6114	FELTSMKYF	HLA-B*18:01, HLA-B*44:03	-0.10515
	6116	LTSMKYFVK	HLA-A*11:01	-0.11765
	6121	YFVKIGPER	HLA-A*33:03	**0.08418**
	6143	TASDTYACW	HLA-B*58:01	**0.22081**
	6147	TYACWHHSI	HLA-A*24:02, HLA-A*24:07	**0.23373**
	6153	HSIGFDYVY	HLA-A*34:01, HLA-B*15:02, HLA-B*15:13, HLA-B*15:21, HLA-B*18:01, HLA-B*35:05, HLA-B*58:01	-0.21438
	6156	GFDYVYNPF	HLA-A*24:02	**0.01657**
	6158	DYVYNPFMI	HLA-A*24:02, HLA-A*24:07	**0.56221**
	6169	QQWGFTGNL	HLA-B*38:02	**0.28654**
	6194	ASCDAIMTR	HLA-A*11:01	-0.01991
	6205	AVHECFVKR	HLA-A*33:03	-0.13895
	6237	QHMVVKAAL	HLA-B*38:02	**0.14003**
	6242	KAALLADKF	HLA-B*58:01	-0.29399
	6244	ALLADKFPV	HLA-A*02:01	-0.34559
	6245	LLADKFPVL	HLA-A*02:01, HLA-A*02:03	**0.16567**
	6283	KAYKIEELF	HLA-B*58:01	**0.1449**
	6288	EELFYSYAT	HLA-B*18:01	**0.09637**
	6292	YSYATHSDK	HLA-A*11:01	**0.06738**
	6300	KFTDGVCLF	HLA-A*24:02, HLA-A*24:07	**0.11058**
	6301	FTDGVCLFW	HLA-B*58:01	**0.10048**
	6320	SIVCRFDTR	HLA-A*33:03	**0.17586**

	6342	SLYVNKHAF	HLA-B*15:02, HLA-B*15:21	**0.0538**
	6352	TPAFDKSAF	HLA-B*35:05	**0.02527**
	6360	FVNLKQLPF	HLA-B*15:02, HLA-B*15:13, HLA-B*15:21, HLA-B*35:05	**0.10244**
	6417	LYLDAYNMM	HLA-A*24:02, HLA-A*24:07	**0.31503**
	6418	YLDAYNMMI	HLA-A*02:01, HLA-A*02:03	-0.27361
	6424	MMISAGFSL	HLA-A*02:01, HLA-A*02:03, HLA-B*15:02, HLA-B*15:13, HLA-B*15:21, HLA-B*35:05, HLA-B*38:02	**0.38891**
	6425	MISAGFSLW	HLA-B*15:13, HLA-B*58:01	**0.08916**
	6428	AGFSLWVYK	HLA-A*11:01	**0.29717**
	6433	WVYKQFDTY	HLA-A*34:01, HLA-B*15:02, HLA-B*15:13, HLA-B*15:21, HLA-B*35:05	**0.06486**
	6436	KQFDTYNLW	HLA-A*24:02, HLA-A*24:07, HLA-B*44:03, HLA-B*58:01	**0.00709**
	6439	DTYNLWNTF	HLA-A*34:01, HLA-B*15:13	-0.00081
	6441	YNLWNTFTR	HLA-A*33:03	-0.1794
	6453	LENVAFNVV	HLA-B*18:01, HLA-B*44:03	**0.0664**
	6455	NVAFNVVNK	HLA-A*34:01	**0.04889**
	6477	IINNTVYTK	HLA-A*11:01	**0.0645**
	6494	FENKTTLPV	HLA-B*18:01, HLA-B*38:02, HLA-B*44:03	**0.08616**
	6498	TTLPVNVAF	HLA-A*24:07, HLA-B*15:13, HLA-B*35:05, HLA-B*58:01	-0.24291
	6500	LPVNVAFEL	HLA-B*35:05, HLA-B*38:02	**0.19663**
	6504	VAFELWAKR	HLA-A*33:03	-0.16048
	6531	AANTVIWDY	HLA-B*58:01	**0.08778**
	6533	NTVIWDYKR	HLA-A*33:03	**0.00704**
	6553	CSMTDIAKK	HLA-A*11:01	-0.05636
	6564	ETICAPLTV	HLA-A*34:01	-0.06071
	6621	EAVKTQFNY	HLA-A*34:01, HLA-B*35:05	-0.0084
	6624	KTQFNYYKK	HLA-A*11:01	**0.13481**
	6652	QEFKPRSQM	HLA-B*18:01	**0.02869**
	6670	DEFIERYKL	HLA-B*18:01	-0.02964
	6673	IERYKLEGY	HLA-B*18:01	**0.00481**
	6683	FEHIVYGDF	HLA-B*18:01, HLA-B*44:03	-0.19311
	6694	SQLGGLHLL	HLA-A*02:01, HLA-B*38:02	**0.09274**
	6700	HLLIGLAKR	HLA-A*33:03	-0.21392
	6708	RFKESPFEL	HLA-A*24:02, HLA-A*24:07	**0.08919**
	6714	FELEDFIPM	HLA-B*18:01, HLA-B*38:02, HLA-B*44:03	**0.16174**
	6748	LLLDDFVEI	HLA-A*02:01, HLA-A*02:03	-0.00382
	6749	LLDDFVEII	HLA-A*02:01	-0.07934
	6775	YTEISFMLW	HLA-B*58:01	**0.0762**
	6781	MLWCKDGHV	HLA-A*02:01, HLA-A*02:03	-0.30072
	6800	QAWQPGVAM	HLA-B*15:02, HLA-B*15:21, HLA-B*35:05	-0.00467

	6805	GVAMPNLYK	HLA-A*11:01	-0.26781
	6806	VAMPNLYKM	HLA-B*58:01	-0.26781
	6808	MPNLYKMQR	HLA-A*33:03	**0.24316**
	6813	KMQRMLLEK	HLA-A*11:01	**0.01876**
	6819	LEKCDLQNY	HLA-B*18:01, HLA-B*44:03	**0.37101**
	6843	KYTQLCQYL	HLA-A*24:02, HLA-A*24:07	-0.06866
	6848	CQYLNTLTL	HLA-B*38:02	**0.00131**
	6850	YLNTLTLAV	HLA-A*02:01, HLA-A*02:03	-0.38452
	6854	LTLAVPYNM	HLA-B*58:01	**0.02327**
	6855	TLAVPYNMR	HLA-A*33:03	**0.03262**
	6885	WLPTGTLLV	HLA-A*02:01, HLA-A*02:03	-0.00472
	6900	FVSDADSTL	HLA-A*34:01	-0.29512
	6907	TLIGDCATV	HLA-A*02:01, HLA-A*02:03	-0.29512
	6913	ATVHTANKW	HLA-B*58:01	-0.02024
	6941	DSKEGFFTY	HLA-B*18:01	-0.11632
	6969	TEHSWNADL	HLA-B*38:02, HLA-B*44:03	-0.03916
	6971	HSWNADLYK	HLA-A*11:01	**0.02621**
	6978	YKLMGHFAW	HLA-B*18:01	-0.21969
	6979	KLMGHFAWW	HLA-B*58:01	**0.06492**
	6982	GHFAWWTAF	HLA-B*38:02	**0.22651**
	7000	EAFLIGCNY	HLA-A*34:01, HLA-B*15:02, HLA-B*15:13, HLA-B*15:21, HLA-B*35:05	**0.07781**
	7013	REQIDGYVM	HLA-B*44:03	**0.0822**
	7019	YVMHANYIF	HLA-A*24:02, HLA-A*24:07, HLA-B*15:02, HLA-B*15:13, HLA-B*15:21, HLA-B*35:05	-0.12171
	7020	VMHANYIFW	HLA-A*24:07, HLA-B*58:01	-0.25594
	7021	MHANYIFWR	HLA-A*33:03	-0.02099
	7026	IFWRNTNPI	HLA-A*24:02, HLA-A*24:07	**0.00222**
	7028	WRNTNPIQL	HLA-B*38:02	-0.41327
	7034	IQLSSYSLF	HLA-A*24:02, HLA-A*24:07	-0.11819
	7039	YSLFDMSKF	HLA-B*15:13	**0.00966**
	7039	YSLFDMSKF	HLA-B*58:01	-0.17256
	7044	MSKFPLKLR	HLA-A*33:03	-0.11517
ORF2 (S)	1	FVFLVLLPL	HLA-A*02:01, HLA-A*34:01	**0.04076**
	23	LPPAYTNSF	HLA-B*35:05	-0.03341
	35	VYYPDKVFR	HLA-A*33:03	-0.09052
	55	LPFFSNVTW	HLA-B*15:13, HLA-B*35:05	**0.04613**
	83	LPFNDGVYF	HLA-B*15:02, HLA-B*15:13, HLA-B*15:21, HLA-B*18:01, HLA-B*35:05	**0.11767**
	88	GVYFASTEK	HLA-A*11:01	**0.09023**

	143	YYHKNNKSW	HLA-A*24:02, HLA-A*24:07	-0.49755
	151	WMESEFRVY	HLA-B*15:02, HLA-B*15:21, HLA-B*35:05	**0.14153**
	159	YSSANNCTF	HLA-A*24:07, HLA-B*15:02, HLA-B*15:13, HLA-B*15:21, HLA-B*35:05, HLA-B*58:01	-0.04954
	161	SANNCTFEY	HLA-A*11:01, HLA-B*15:02, HLA-B*15:13, HLA-B*15:21, HLA-B*35:05, HLA-B*58:01	**0.13273**
	166	TFEYVSQPF	HLA-A*24:02, HLA-A*24:07	-0.19099
	167	FEYVSQPFL	HLA-B*18:01, HLA-B*38:02, HLA-B*44:03	-0.17076
	191	FVFKNIDGY	HLA-A*34:01, HLA-B*15:21	-0.0215
	192	VFKNIDGYF	HLA-A*24:02	**0.10041**
	201	KIYSKHTPI	HLA-A*02:03	-0.32094
	235	TRFQTLLAL	HLA-B*38:02	-0.0377
	239	TLLALHRSY	HLA-B*15:02, HLA-B*15:21	**0.00244**
	240	LLALHRSYL	HLA-A*02:03	-0.06002
	257	WTAGAAAYY	HLA-A*34:01, HLA-B*58:01	**0.15259**
	264	YYVGYLQPR	HLA-A*33:03	-0.07078
	268	YLQPRTFLL	HLA-A*02:01, HLA-A*02:03, HLA-A*24:02, HLA-A*24:07, HLA-B*38:02	**0.1305**
	301	TLKSFTVEK	HLA-A*11:01, HLA-A*33:03	**0.00741**
	311	IYQTSNFRV	HLA-A*24:02, HLA-A*24:07	-0.03669
	320	QPTESIVRF	HLA-B*35:05	**0.14261**
	338	GEVFNATRF	HLA-B*44:03	**0.22473**
	342	NATRFASVY	HLA-A*34:01, HLA-B*15:21	**0.10001**
	344	TRFASVYAW	HLA-B*38:02, HLA-B*44:03	-0.02513
	346	FASVYAWNR	HLA-A*33:03	**0.20423**
	348	SVYAWNRKR	HLA-A*33:03	**0.16546**
	360	CVADYSVLY	HLA-A*11:01, HLA-A*34:01, HLA-B*15:21	-0.09595
	365	SVLYNSASF	HLA-B*15:02, HLA-B*15:13, HLA-B*15:21, HLA-B*35:05	-0.23299
	368	YNSASFSTF	HLA-A*24:02, HLA-A*24:07, HLA-B*15:02, HLA-B*15:13 HLA-B*15:21, HLA-B*35:05	-0.18217
	369	NSASFSTFK	HLA-A*11:01	-0.09434
	371	ASFSTFKCY	HLA-B*15:02	-0.19397
	387	NDLCFTNVY	HLA-B*18:01	**0.11135**
	391	FTNVYADSF	HLA-B*15:13, HLA-B*58:01	-0.00527
	416	KIADYNYKL	HLA-A*02:01	-0.10379
	447	NYNYLYRLF	HLA-A*24:02	**0.0171**
	448	YNYLYRLFR	HLA-A*33:03	**0.0918**
	449	NYLYRLFRK	HLA-A*33:03	**0.16168**
	453	RLFRKSNLK	HLA-A*11:01	-0.28759
	457	KSNLKPFER	HLA-A*33:03	-0.0764
	463	FERDISTEI	HLA-B*44:03	**0.10425**

	488	YFPLQSYGF	HLA-A*24:02, HLA-A*24:07	-0.26661
	504	YQPYRVVVL	HLA-B*38:02	**0.1409**
	506	PYRVVVLSF	HLA-A*24:02, HLA-A*24:07	**0.03138**
	553	ESNKKFLPF	HLA-B*15:02, HLA-B*15:13	-0.33474
	553	ESNKKFLPF	HLA-B*15:21	-0.33474
	558	FLPFQQFGR	HLA-A*33:03	**0.01096**
	567	DIADTTDAV	HLA-A*34:01	**0.15094**
	603	TSNQVAVLY	HLA-B*58:01	-0.01327
	624	HADQLTPTW	HLA-B*58:01	-0.0703
	627	QLTPTWRVY	HLA-B*15:02, HLA-B*15:21	**0.31555**
	634	VYSTGSNVF	HLA-A*24:02, HLA-A*24:07	-0.11871
	637	TGSNVFQTR	HLA-A*33:03	**0.01511**
	676	QTNSPRRAR	HLA-A*33:03	-0.06411
	686	VASQSIIAY	HLA-B*15:02, HLA-B*15:13, HLA-B*15:21, HLA-B*35:05, HLA-B*58:01	-0.0709
	688	SQSIIAYTM	HLA-B*38:02	**0.26621**
	690	SIIAYTMSL	HLA-A*02:01, HLA-A*02:03, HLA-A*34:01, HLA-B*15:21, HLA-B*38:02	-0.12935
	698	LGAENSVAY	HLA-B*15:02, HLA-B*15:21, HLA-B*35:05	**0.00912**
	711	IAIPTNFTI	HLA-A*24:02, HLA-A*24:07, HLA-B*15:13, HLA-B*58:01	**0.18523**
	713	IPTNFTISV	HLA-B*35:05	**0.17229**
	717	FTISVTTEI	HLA-A*02:01, HLA-A*02:03, HLA-A*34:01	**0.04473**
	724	EILPVSMTK	HLA-A*11:01, HLA-A*34:01	-0.25581
	732	KTSVDCTMY	HLA-B*58:01	-0.11115
	733	TSVDCTMYI	HLA-B*58:01	-0.1306
	756	GSFCTQLNR	HLA-A*11:01	-0.10063
	761	QLNRALTGI	HLA-A*02:03	**0.1302**
	814	RSFIEDLLF	HLA-B*58:01	**0.27446**
	820	LLFNKVTLA	HLA-A*02:01, HLA-A*02:03	-0.11337
	826	TLADAGFIK	HLA-A*11:01	**0.28158**
	860	LPPLLTDEM	HLA-B*35:05	**0.0882**
	868	MIAQYTSAL	HLA-A*02:03, HLA-A*34:01, HLA-B*15:02, HLA-B*15:21, HLA-B*35:05	-0.18768
	877	LAGTITSGW	HLA-B*58:01	**0.09638**
	879	GTITSGWTF	HLA-A*24:02, HLA-A*24:07, HLA-B*15:13, HLA-B*58:01	**0.16268**
	885	WTFGAGAAL	HLA-A*34:01, HLA-B*15:21	**0.19798**
	893	LQIPFAMQM	HLA-B*38:02	-0.03301
	895	IPFAMQMAY	HLA-B*15:02, HLA-B*15:13, HLA-B*15:21, HLA-B*18:01, HLA-B*35:05	-0.32801
	897	FAMQMAYRF	HLA-B*15:13, HLA-B*35:05, HLA-B*58:01	-0.28061
	938	SSTASALGK	HLA-A*11:01	-0.06186

	974	SVLNDILSR	HLA-A*11:01	**0.03075**
	975	VLNDILSRL	HLA-A*02:01, HLA-A*02:03	**0.03**
	982	RLDKVEAEV	HLA-A*02:01	**0.01617**
	988	AEVQIDRLI	HLA-B*44:03	**0.08452**
	999	RLQSLQTYV	HLA-A*02:01, HLA-A*02:03, HLA-B*44:03	-0.29331
	1015	AEIRASANL	HLA-B*44:03	**0.00689**
	1019	ASANLAATK	HLA-A*11:01	**0.08792**
	1059	VVFLHVTYV	HLA-A*02:01, HLA-A*02:03, HLA-A*34:01	**0.1278**
	1061	FLHVTYVPA	HLA-A*02:03	**0.11472**
	1064	VTYVPAQEK	HLA-A*11:01	**0.02711**
	1094	FVSNGTHWF	HLA-B*15:1, HLA-B*15:21	**0.16605**
	1098	GTHWFVTQR	HLA-A*11:01, HLA-A*33:03	**0.35133**
	1112	QIITTDNTF	HLA-B*15:02, HLA-B*15:21	**0.15816**
	1200	QELGKYEQY	HLA-B*18:01, HLA-B*44:03	-0.16616
	1205	YEQYIKWPW	HLA-B*18:01, HLA-B*44:03	**0.06574**
	1207	QYIKWPWYI	HLA-A*24:02, HLA-A*24:07	**0.21624**
	1208	YIKWPWYIW	HLA-B*58:01	**0.42524**
	1211	WPWYIWLGF	HLA-B*18:01, HLA-B*35:05	**0.41673**
	1219	FIAGLIAIV	HLA-A*02:01, HLA-A*02:03	**0.27206**
	1236	MTSCCSCLK	HLA-A*11:01, HLA-A*33:03	-0.36816
ORF4	15	SVLLFLAFV	HLA-A*02:01	**0.19022**
	17	LLFLAFVVF	HLA-B*15:02, HLA-B*15:13, HLA-B*15:21	**0.2341**
	19	FLAFVVFLL	HLA-A*02:01, HLA-A*02:03	**0.30188**
	25	FLLVTLAIL	HLA-A*02:01	**0.17608**
	29	TLAILTALR	HLA-A*33:03	**0.1989**
	33	LTALRLCAY	HLA-B*15:02, HLA-B*15:21	**0.01886**
	49	SLVKPSFYV	HLA-A*02:01, HLA-A*02:03	-0.27349
	50	LVKPSFYVY	HLA-B*15:02, HLA-B*15:13, HLA-B*15:21, HLA-B*35:05	-0.11106
	60	RVKNLNSSR	HLA-A*33:03	
ORF5	14	KLLEQWNLV	HLA-A*02:01	**0.18092**
	25	FLFLTWICL	HLA-A*02:01	**0.35397**
	28	LTWICLLQF	HLA-B*58:01	**0.06584**
	33	LLQFAYANR	HLA-A*33:03	**0.14406**
	36	FAYANRNRF	HLA-B*15:13	**0.10537**
	38	YANRNRFLY	HLA-B*35:05	**0.18472**
	42	NRFLYIIKL	HLA-B*38:02	**0.13484**
	46	YIIKLIFLW	HLA-B*58:01	**0.033**
	49	KLIFLWLLW	HLA-B*58:01	**0.34287**
	53	LWLLWPVTL	HLA-A*24:02, HLA-A*24:07	**0.24802**
	54	WLLWPVTLA	HLA-A*02:01	**0.27363**

	56	LWPVTLACF	HLA-A*24:02, HLA-A*24:07	**0.06682**
	64	FVLAAVYRI	HLA-A*02:01	**0.13985**
	66	LAAVYRINW	HLA-B*58:01	**0.2079**
	83	MACLVGLMW	HLA-B*58:01	-0.06852
	88	GLMWLSYFI	HLA-A*02:01, HLA-A*02:03	**0.06464**
	91	WLSYFIASF	HLA-B*15:02, HLA-B*15:13, HLA-B*15:21	**0.11822**
	92	LSYFIASFR	HLA-A*11:01, HLA-A*33:03	**0.21181**
	93	SYFIASFRL	HLA-A*24:02, HLA-A*24:07	**0.18333**
	94	YFIASFRLF	HLA-A*24:02, HLA-A*24:07	**0.06887**
	96	IASFRLFAR	HLA-A*33:03	**0.22572**
	100	RLFARTRSM	HLA-B*15:02, HLA-B*15:21	**0.11133**
	101	LFARTRSMW	HLA-A*24:02, HLA-A*24:07	-0.09092
	135	SELVIGAVI	HLA-B*44:03	**0.25658**
	169	VATSRTLSY	HLA-B*15:02, HLA-B*15:13, HLA-B*15:21, HLA-B*35:05, HLA-B*58:01	-0.17295
	170	ATSRTLSYY	HLA-A*11:01, HLA-A*34:01, HLA-B*58:01	-0.11604
	171	TSRTLSYYK	HLA-A*11:01	-0.11595
	177	YYKLGASQR	HLA-A*33:03	-0.21863
	195	YSRYRIGNY	HLA-A*34:01, HLA-B*15:02, HLA-B*15:13, HLA-B*15:21	**0.21358**
	197	RYRIGNYKL	HLA-A*24:02, HLA-A*24:07	**0.04851**
ORF9	44	LPNNTASWF	HLA-B*35:05	**0.05582**
	47	NTASWFTAL	HLA-A*34:01, HLA-B*15:21	**0.22775**
	65	FPRGQGVPI	HLA-B*35:05	-0.00164
	78	SPDDQIGYY	HLA-B*35:05	**0.06844**
	83	IGYYRRATR	HLA-A*33:03	**0.1499**
	85	YYRRATRRI	HLA-A*24:02<br>HLA-A*24:07	**0.21744**
	99	KMKDLSPRW	HLA-B*58:01	-0.19333
	102	DLSPRWYFY	HLA-A*34:01	**0.25933**
	103	LSPRWYFYY	HLA-A*24:07, HLA-B*58:01	**0.35734**
	123	GANKDGIIW	HLA-B*58:01	**0.02448**
	180	QASSRSSSR	HLA-A*33:03	-0.56178
	186	SSRSRNSSR	HLA-A*33:03	-0.34164
	221	LLLDRLNQL	HLA-A*02:01, HLA-A*02:03	-0.01446
	265	KAYNVTQAF	HLA-A*24:02, HLA-A*24:07, HLA-B*15:02, HLA-B*15:13, HLA-B*15:21, HLA-B*35:05, HLA-B*58:01	-0.00587
	268	NVTQAFGRR	HLA-A*33:03	**0.10318**
	298	KHWPQIAQF	HLA-A*24:02, HLA-A*24:07, HLA-B*38:02	**0.03856**
	306	FAPSASAFF	HLA-B*15:13, HLA-B*35:05	-0.18628
	310	ASAFFGMSR	HLA-A*11:01	**0.03154**

	315	GMSRIGMEV	HLA-A*02:03	**0.07018**
	321	MEVTPSGTW	HLA-B*15:13, HLA-B*18:01, HLA-B*44:03	-0.06279
	322	EVTPSGTWL	HLA-A*34:01	**0.03442**
	324	TPSGTWLTY	HLA-B*15:21, HLA-B*18:01, HLA-B*35:05	**0.24003**
	328	TWLTYTGAI	HLA-A*24:07	**0.11986**
	351	LLNKHIDAY	HLA-B*15:02, HLA-B*15:21, HLA-B*35:05	-0.02074
	354	KHIDAYKTF	HLA-A*24:02	-0.05918
	360	KTFPPTEPK	HLA-A*11:01	**0.1306**
	394	LPAADLDDF	HLA-B*35:05	**0.09491**

Table S2. Full list of predicted 15-mer HTL epitopes from five different SARS-CoV-2 proteins (ORF1ab, S, E, M, and N) with %Rank less than 1% identified from NetMHCpan 4.0 along with their IFN-γ score. A total of 339 promiscuous peptides (bind to more than one different HLA alleles) out of 1,422 predicted peptides were identified and indicated by green color highlight. A total of 875 peptides with positive IFN-γ scores out of 1,422 were identified and highlighted by bold-colored text.

Protein	Start Residue	Peptide	HLA Class II Allele	IFN-γ score
ORF1ab	16	LSLPVLQVRDVLVRG	HLA DRB1*15:01	-0.552587
	17	SLPVLQVRDVLVRGF	HLA DRB1*15:01	-0.50274156
	62	PQLEQPYVFIKRSDA	HLA DRB1*11:01	-0.37322428
	63	QLEQPYVFIKRSDAR	HLA DRB1*11:01	-0.066575101
	64	LEQPYVFIKRSDART	HLA DRB1*11:01, HLA DRB1*12:02	-0.1160575
	65	EQPYVFIKRSDARTA	HLA DRB1*11:01, HLA DRB1*12:02	-0.18932492
	66	QPYVFIKRSDARTAP	HLA DRB1*11:01	-0.16594135
	67	PYVFIKRSDARTAPH	HLA DRB1*11:01	-0.2621495
	85	MVELVAELEGIQYGR	HLA DRB1*12:02	**0.22164066**
	86	VELVAELEGIQYGRS	HLA DRB1*12:02	**0.21184544**
	170	TRELMRELNGGAYTR	HLA DRB1*12:02	**0.09058035**
	171	RELMRELNGGAYTRY	HLA DRB1*12:02	**0.079825801**
	178	NGGAYTRYVDNNFCG	HLA DRB1*15:02	**0.028698538**
	179	GGAYTRYVDNNFCGP	HLA DRB1*15:02	-0.33259633
	244	RSEKSYELQTPFEIK	HLA DRB1*07:01	**0.1123738**
	245	SEKSYELQTPFEIKL	HLA DRB1*07:01, HLA DRB1*16:02	**0.11812166**
	246	EKSYELQTPFEIKLA	HLA DRB1*07:01, HLA DRB1*16:02	**0.14008837**
	273	FVFPLNSIIKTIQPR	HLA DRB1*11:01	-0.25530678
	274	VFPLNSIIKTIQPRV	HLA DRB1*11:01	-0.24941483
	276	PLNSIIKTIQPRVEK	HLA DRB1*12:02	-0.15153035
	277	LNSIIKTIQPRVEKK	HLA DRB1*12:02	-0.25481012
	278	NSIIKTIQPRVEKKK	HLA DRB1*12:02	-0.18888334
	379	GPEHSLAEYHNESGL	HLA DRB1*15:01	-0.51376695
	380	PEHSLAEYHNESGLK	HLA DRB1*15:01, HLA DRB1*15:02	-0.50054293
	381	EHSLAEYHNESGLKT	HLA DRB1*15:01, HLA DRB1*15:02	-0.31709863
	382	HSLAEYHNESGLKTI	HLA DRB1*15:01	-0.24287768
	388	HNESGLKTILRKGGR	HLA DRB1*11:01	**0.14848681**
	389	NESGLKTILRKGGRT	HLA DRB1*11:01	**0.43200233**
	390	ESGLKTILRKGGRTI	HLA DRB1*11:01	**0.50723971**
	391	SGLKTILRKGGRTIA	HLA DRB1*11:01	**0.67477291**
	446	LNDNLLEILQKEKVN	HLA DRB1*12:02	-0.28006885
	447	NDNLLEILQKEKVNI	HLA DRB1*12:02	**0.13110528**
	448	DNLLEILQKEKVNIN	HLA DRB1*11:01, HLA DRB1*12:02	**0.055569224**
	449	NLLEILQKEKVNINI	HLA DRB1*12:02	**0.26354633**
	456	KEKVNINIVGDFKLN	HLA DRB1*15:01, HLA DRB1*15:02, HLA DRB1*16:02	-0.10097192

	457	EKVNINIVGDFKLNE	HLA DRB1*12:02, HLA DRB1*15:01, HLA DRB1*15:02, HLA DRB1*16:02	**0.073961117**
	458	KVNINIVGDFKLNEE	HLA DRB1*12:02, HLA DRB1*15:01, HLA DRB1*15:02, HLA DRB1*16:02	-0.10696324
	459	VNINIVGDFKLNEEI	HLA DRB1*15:01, HLA DRB1*15:02, HLA DRB1*16:02	-0.033490438
	475	IILASFSASTSAFVE	HLA DRB1*07:01	**0.48889866**
	476	ILASFSASTSAFVET	HLA DRB1*07:01	**0.37138483**
	477	LASFSASTSAFVETV	HLA DRB1*07:01	**0.41160771**
	478	ASFSASTSAFVETVK	HLA DRB1*07:01	**0.19966591**
	483	STSAFVETVKGLDYK	HLA DRB1*11:01	**0.18968108**
	484	TSAFVETVKGLDYKA	HLA DRB1*11:01, HLA DRB1*12:02	**0.21531222**
	485	SAFVETVKGLDYKAF	HLA DRB1*12:02	-0.012206208
	527	KSILSPLYAFASEAA	HLA DRB1*12:02	-0.2415999
	528	SILSPLYAFASEAAR	HLA DRB1*15:01, HLA DRB1*15:02, HLA DRB1*16:02	**0.17674965**
	529	ILSPLYAFASEAARV	HLA DRB1*15:01, HLA DRB1*15:02, HLA DRB1*16:02	**0.033121519**
	530	LSPLYAFASEAARVV	HLA DRB1*15:01, HLA DRB1*15:02, HLA DRB1*16:02	**0.024506055**
	531	SPLYAFASEAARVVR	HLA DRB1*15:01, HLA DRB1*15:02, HLA DRB1*16:02	**0.36250218**
	532	PLYAFASEAARVVRS	HLA DRB1*07:01, HLA DRB1*16:02	**0.29254928**
	541	ARVVRSIFSRTLETA	HLA DRB1*12:02	**0.48722758**
	553	ETAQNSVRVLQKAAI	HLA DRB1*12:02	-0.23633119
	554	TAQNSVRVLQKAAIT	HLA DRB1*12:02	**0.068440439**
	555	AQNSVRVLQKAAITI	HLA DRB1*12:02	-0.027888451
	556	QNSVRVLQKAAITIL	HLA DRB1*12:02	-0.018083756
	557	NSVRVLQKAAITILD	HLA DRB1*12:02	-0.17235933
	563	QKAAITILDGISQYS	HLA DRB1*12:02	-0.34942023
	564	KAAITILDGISQYSL	HLA DRB1*12:02	-0.50168694
	593	NNLVVMAYITGGVVQ	HLA DRB1*15:01	-0.41352632
	601	ITGGVVQLTSQWLTN	HLA DRB1*15:01, HLA DRB1*15:02, HLA DRB1*16:02	-0.3300186
	602	TGGVVQLTSQWLTNI	HLA DRB1*16:02	-0.32121582
	674	SVQTFFKLVNKFLAL	HLA DRB1*11:01	-0.58133375
	675	VQTFFKLVNKFLALC	HLA DRB1*11:01	-0.2932627
	676	QTFFKLVNKFLALCA	HLA DRB1*11:01	-0.33515995
	714	KGLYRKCVKSREETG	HLA DRB1*11:01	-0.3148611
	734	KAPKEIIFLEGETLP	HLA DRB1*12:02, HLA DRB1*15:01, HLA DRB1*15:02, HLA DRB1*16:02	-0.24929914
	735	APKEIIFLEGETLPT	HLA DRB1*12:02, HLA DRB1*15:01, HLA DRB1*15:02, HLA DRB1*16:02	**0.082149378**
	736	PKEIIFLEGETLPTE	HLA DRB1*12:02, HLA DRB1*15:01, HLA DRB1*15:02, HLA DRB1*16:02	**0.077069314**
	737	KEIIFLEGETLPTEV	HLA DRB1*12:02, HLA DRB1*15:02, HLA DRB1*16:02	**0.51846126**
	827	DDTVIEVQGYKSVNI	HLA DRB1*15:01	-0.2319852

	828	DTVIEVQGYKSVNIT	HLA DRB1*15:01, HLA DRB1*15:02	-0.25329015
	829	TVIEVQGYKSVNITF	HLA DRB1*15:01, HLA DRB1*15:02, HLA DRB1*16:02	-0.31288866
	830	VIEVQGYKSVNITFE	HLA DRB1*15:01, HLA DRB1*15:02, HLA DRB1*16:02	-0.16164877
	831	IEVQGYKSVNITFEL	HLA DRB1*15:01, HLA DRB1*15:02	-0.41585405
	832	EVQGYKSVNITFELD	HLA DRB1*15:01	-0.2633236
	958	KPLEFGATSAALQPE	HLA DRB1*07:01	-0.66065581
	959	PLEFGATSAALQPEE	HLA DRB1*07:01	-0.61293064
	1027	SFSGYLKLTDNVYIK	HLA DRB1*07:01	**0.075951363**
	1028	FSGYLKLTDNVYIKN	HLA DRB1*07:01, HLA DRB1*15:02	-0.20400283
	1029	SGYLKLTDNVYIKNA	HLA DRB1*07:01, HLA DRB1*15:02	-0.26796184
	1052	VKPTVVVNAANVYLK	HLA DRB1*12:02, HLA DRB1*15:01, HLA DRB1*15:02	-0.37547334
	1053	KPTVVVNAANVYLKH	HLA DRB1*12:02, HLA DRB1*15:01, HLA DRB1*15:02, HLA DRB1*16:02	-0.45376782
	1054	PTVVVNAANVYLKHG	HLA DRB1*15:01, HLA DRB1*15:02, HLA DRB1*16:02	**0.091724496**
	1055	TVVVNAANVYLKHGG	HLA DRB1*15:01	-0.052916021
	1086	ESDDYIATNGPLKVG	HLA DRB1*16:02	-0.27560246
	1087	SDDYIATNGPLKVGG	HLA DRB1*16:02	-0.31800246
	1123	NKGEDIQLLKSAYEN	HLA DRB1*12:02	-0.2003168
	1124	KGEDIQLLKSAYENF	HLA DRB1*12:02	-0.34475917
	1125	GEDIQLLKSAYENFN	HLA DRB1*12:02, HLA DRB1*15:01, HLA DRB1*16:02	-0.41469217
	1126	EDIQLLKSAYENFNQ	HLA DRB1*12:02	-0.41980237
	1170	RTNVYLAVFDKNLYD	HLA DRB1*16:02	-0.32634074
	1171	TNVYLAVFDKNLYDK	HLA DRB1*07:01, HLA DRB1*12:02, HLA DRB1*15:02, HLA DRB1*16:02	-0.32342333
	1172	NVYLAVFDKNLYDKL	HLA DRB1*12:02	-0.30781439
	1186	LVSSFLEMKSEKQVE	HLA DRB1*11:01	-0.10828218
	1187	VSSFLEMKSEKQVEQ	HLA DRB1*11:01, HLA DRB1*16:02	**0.089900492**
	1198	QVEQKIAEIPKEEVK	HLA DRB1*12:02	-0.88669915
	1199	VEQKIAEIPKEEVKP	HLA DRB1*11:01, HLA DRB1*12:02	-0.94164818
	1200	EQKIAEIPKEEVKPF	HLA DRB1*11:01, HLA DRB1*12:02	-0.78571588
	1210	EVKPFITESKPSVEQ	HLA DRB1*11:01	**0.035338064**
	1211	VKPFITESKPSVEQR	HLA DRB1*11:01	**0.3156851**
	1248	FLTENLLLYIDINGN	HLA DRB1*15:01	-0.055555446
	1249	LTENLLLYIDINGNL	HLA DRB1*15:01	**0.028326154**
	1250	TENLLLYIDINGNLH	HLA DRB1*15:01	-0.27724578
	1295	VLTAVVIPTKKAGGT	HLA DRB1*11:01	**0.23158712**
	1296	LTAVVIPTKKAGGTT	HLA DRB1*11:01	**0.18044431**
	1297	TAVVIPTKKAGGTTE	HLA DRB1*11:01	**0.02444769**
	1298	AVVIPTKKAGGTTEM	HLA DRB1*11:01	-0.28936136
	1320	VPTDNYITTYPGQGL	HLA DRB1*15:01, HLA DRB1*15:02	-0.22405757

	1321	PTDNYITTYPGQGLN	HLA DRB1*15:01, HLA DRB1*15:02, HLA DRB1*16:02	-0.043747134
	1322	TDNYITTYPGQGLNG	HLA DRB1*15:01, HLA DRB1*15:02, HLA DRB1*16:02	-0.20247955
	1323	DNYITTYPGQGLNGY	HLA DRB1*15:01, HLA DRB1*15:02, HLA DRB1*16:02	-0.0016888477
	1324	NYITTYPGQGLNGYT	HLA DRB1*15:01, HLA DRB1*15:02, HLA DRB1*16:02	-0.26969085
	1325	YITTYPGQGLNGYTV	HLA DRB1*15:01, HLA DRB1*15:02	-0.17061748
	1333	GLNGYTVEEAKTVLK	HLA DRB1*16:02	-0.093713228
	1334	LNGYTVEEAKTVLKK	HLA DRB1*16:02	-0.40134505
	1335	NGYTVEEAKTVLKKC	HLA DRB1*16:02	-0.21117347
	1348	KCKSAFYILPSIISN	HLA DRB1*11:01, HLA DRB1*15:02, HLA DRB1*16:02	**0.24456538**
	1349	CKSAFYILPSIISNE	HLA DRB1*11:01, HLA DRB1*12:02, HLA DRB1*15:02, HLA DRB1*16:02	**0.28976547**
	1350	KSAFYILPSIISNEK	HLA DRB1*11:01, HLA DRB1*12:02, HLA DRB1*15:02, HLA DRB1*16:02	**0.33784834**
	1351	SAFYILPSIISNEKQ	HLA DRB1*11:01, HLA DRB1*15:02, HLA DRB1*16:02	**0.088032125**
	1362	NEKQEILGTVSWNLR	HLA DRB1*07:01	**0.037044045**
	1363	EKQEILGTVSWNLRE	HLA DRB1*07:01, HLA DRB1*15:01, HLA DRB1*15:02, HLA DRB1*16:02	-0.0096068911
	1364	KQEILGTVSWNLREM	HLA DRB1*07:01, HLA DRB1*15:01, HLA DRB1*15:02	-0.1963119
	1365	QEILGTVSWNLREML	HLA DRB1*07:01	**0.031508802**
	1393	VETKAIVSTIQRKYK	HLA DRB1*12:02	-0.50582647
	1394	ETKAIVSTIQRKYKG	HLA DRB1*12:02	-0.72880984
	1395	TKAIVSTIQRKYKGI	HLA DRB1*12:02	-0.15436648
	1396	KAIVSTIQRKYKGIK	HLA DRB1*12:02	-0.10905096
	1402	IQRKYKGIKIQEGVV	HLA DRB1*11:01	**0.15369216**
	1403	QRKYKGIKIQEGVVD	HLA DRB1*11:01	**0.11040284**
	1411	IQEGVVDYGARFYFY	HLA DRB1*15:01	**0.1137742**
	1412	QEGVVDYGARFYFYT	HLA DRB1*15:01	**0.031215859**
	1417	DYGARFYFYTSKTTV	HLA DRB1*15:01, HLA DRB1*15:02	-0.46338393
	1418	YGARFYFYTSKTTVA	HLA DRB1*15:01, HLA DRB1*15:02, HLA DRB1*16:02	-0.6194514
	1419	GARFYFYTSKTTVAS	HLA DRB1*07:01, HLA DRB1*15:01, HLA DRB1*15:02, HLA DRB1*16:02	-0.48814565
	1420	ARFYFYTSKTTVASL	HLA DRB1*07:01	-0.39387761
	1420	ARFYFYTSKTTVASL	HLA DRB1*15:02	-0.069073956
	1442	NETLVTMPLGYVTHG	HLA DRB1*12:02	**0.27842636**
	1453	VTHGLNLEEAARYMR	HLA DRB1*12:02	**0.056160056**
	1454	THGLNLEEAARYMRS	HLA DRB1*12:02	-0.10066054
	1459	LEEAARYMRSLKVPA	HLA DRB1*12:02	**0.012642725**
	1460	EEAARYMRSLKVPAT	HLA DRB1*11:01, HLA DRB1*12:02	-0.14505697
	1461	EAARYMRSLKVPATV	HLA DRB1*11:01	**0.043328602**
	1462	AARYMRSLKVPATVS	HLA DRB1*11:01	-0.23289796
	1463	ARYMRSLKVPATVSV	HLA DRB1*11:01	-0.61896465

	1477	VSSPDAVTAYNGYLT	HLA DRB1*15:01	-0.5097373
	1478	SSPDAVTAYNGYLTS	HLA DRB1*15:01, HLA DRB1*15:02	-0.5097373
	1479	SPDAVTAYNGYLTSS	HLA DRB1*15:01, HLA DRB1*15:02, HLA DRB1*16:02	-0.37103342
	1480	PDAVTAYNGYLTSSS	HLA DRB1*15:01, HLA DRB1*15:02, HLA DRB1*16:02	-0.3490795
	1481	DAVTAYNGYLTSSSK	HLA DRB1*15:01, HLA DRB1*15:02	-0.53096363
	1482	AVTAYNGYLTSSSKT	HLA DRB1*15:01	-0.2888732
	1496	TPEEHFIETISLAGS	HLA DRB1*07:01	-0.10822819
	1497	PEEHFIETISLAGSY	HLA DRB1*07:01	-0.070484352
	1498	EEHFIETISLAGSYK	HLA DRB1*07:01, HLA DRB1*11:01, HLA DRB1*16:02	-0.2995449
	1531	GDKSVYYTSNPTTFH	HLA DRB1*15:01	-0.10368188
	1533	KSVYYTSNPTTFHLD	HLA DRB1*07:01	**0.041596604**
	1545	HLDGEVITFDNLKTL	HLA DRB1*15:01, HLA DRB1*15:02	**0.13655355**
	1546	LDGEVITFDNLKTLL	HLA DRB1*15:01, HLA DRB1*15:02	**0.19456278**
	1547	DGEVITFDNLKTLLS	HLA DRB1*15:01, HLA DRB1*15:02	**0.16475137**
	1548	GEVITFDNLKTLLSL	HLA DRB1*15:01	**0.059280899**
	1564	EVRTIKVFTTVDNIN	HLA DRB1*15:01	-0.26678133
	1566	RTIKVFTTVDNINLH	HLA DRB1*16:02	-0.22019882
	1567	TIKVFTTVDNINLHT	HLA DRB1*15:02, HLA DRB1*16:02	-0.18895507
	1568	IKVFTTVDNINLHTQ	HLA DRB1*07:01, HLA DRB1*15:02, HLA DRB1*16:02	-0.24795144
	1569	KVFTTVDNINLHTQV	HLA DRB1*16:02	-0.25357961
	1602	ADVTKIKPHNSHEGK	HLA DRB1*15:01	-0.28325887
	1603	DVTKIKPHNSHEGKT	HLA DRB1*15:01, HLA DRB1*15:02	-0.49109007
	1604	VTKIKPHNSHEGKTF	HLA DRB1*15:01, HLA DRB1*15:02	-0.3614223
	1605	TKIKPHNSHEGKTFY	HLA DRB1*15:01	**0.53212289**
	1630	EAFEYYHTTDPSFLG	HLA DRB1*07:01	**0.7473454**
	1631	AFEYYHTTDPSFLGR	HLA DRB1*07:01	**0.77956564**
	1632	FEYYHTTDPSFLGRY	HLA DRB1*07:01	**0.11555638**
	1694	ALQDAYYRARAGEAA	HLA DRB1*07:01	**0.43996998**
	1695	LQDAYYRARAGEAAN	HLA DRB1*07:01, HLA DRB1*11:01, HLA DRB1*16:02	**0.46088023**
	1696	QDAYYRARAGEAANF	HLA DRB1*07:01, HLA DRB1*11:01, HLA DRB1*16:02	-0.23526409
	1709	NFCALILAYCNKTVG	HLA DRB1*15:01, HLA DRB1*15:02	-0.014200696
	1711	CALILAYCNKTVGEL	HLA DRB1*15:01	-0.34481341
	1712	ALILAYCNKTVGELG	HLA DRB1*15:01	-0.44161946
	1725	LGDVRETMSYLFQHA	HLA DRB1*07:01	**0.042536084**
	1758	QQTTLKGVEAVMYMG	HLA DRB1*12:02	**0.33755187**
	1759	QTTLKGVEAVMYMGT	HLA DRB1*12:02	**0.15259155**
	1764	GVEAVMYMGTLSYEQ	HLA DRB1*12:02	-0.33759888
	1765	VEAVMYMGTLSYEQF	HLA DRB1*12:02	-0.54395245
	1771	MGTLSYEQFKKGVQI	HLA DRB1*11:01	-0.3613913
	1772	GTLSYEQFKKGVQIP	HLA DRB1*11:01	-0.87085008

	1773	TLSYEQFKKGVQIPC	HLA DRB1*11:01	-0.48118012
	1798	VQQESPFVMMSAPPA	HLA DRB1*16:02	-0.2941382
	1799	QQESPFVMMSAPPAQ	HLA DRB1*11:01, HLA DRB1*12:02, HLA DRB1*15:02, HLA DRB1*16:02	-0.31609633
	1800	QESPFVMMSAPPAQY	HLA DRB1*11:01, HLA DRB1*12:02, HLA DRB1*15:02, HLA DRB1*16:02	-0.15478331
	1801	ESPFVMMSAPPAQYE	HLA DRB1*11:01, HLA DRB1*12:02, HLA DRB1*15:02, HLA DRB1*16:02	-0.21040286
	1802	SPFVMMSAPPAQYEL	HLA DRB1*11:01, HLA DRB1*12:02, HLA DRB1*15:02, HLA DRB1*16:02	-0.18691649
	1869	KENSYTTTIKPVTYK	HLA DRB1*11:01	-0.17750343
	1870	ENSYTTTIKPVTYKL	HLA DRB1*11:01	-0.13697578
	1907	FTEQPIDLVPNQPYP	HLA DRB1*12:02, HLA DRB1*15:01	-0.47075197
	1908	TEQPIDLVPNQPYPN	HLA DRB1*12:02, HLA DRB1*15:01, HLA DRB1*15:02, HLA DRB1*16:02	-0.18762311
	1909	EQPIDLVPNQPYPNA	HLA DRB1*12:02, HLA DRB1*15:01, HLA DRB1*15:02, HLA DRB1*16:02	-0.15613773
	1910	QPIDLVPNQPYPNAS	HLA DRB1*12:02, HLA DRB1*15:01	-0.12838139
	1952	SRELKVTFFPDLNGD	HLA DRB1*15:01	-0.75297701
	1953	RELKVTFFPDLNGDV	HLA DRB1*15:01, HLA DRB1*15:02	-0.88047866
	1954	ELKVTFFPDLNGDVV	HLA DRB1*15:01, HLA DRB1*15:02	-0.48028134
	1962	DLNGDVVAIDYKHYT	HLA DRB1*12:02	-0.76428731
	1963	LNGDVVAIDYKHYTP	HLA DRB1*12:02	-0.80127992
	1964	NGDVVAIDYKHYTPS	HLA DRB1*12:02	-0.45343102
	1965	GDVVAIDYKHYTPSF	HLA DRB1*12:02	-0.42693816
	1970	IDYKHYTPSFKKGAK	HLA DRB1*11:01	-0.13972723
	1971	DYKHYTPSFKKGAKL	HLA DRB1*11:01	-0.042158114
	1972	YKHYTPSFKKGAKLL	HLA DRB1*11:01	-0.2310786
	1975	YTPSFKKGAKLLHKP	HLA DRB1*11:01	-0.47902691
	1976	TPSFKKGAKLLHKPI	HLA DRB1*11:01	-0.00052440326
	1985	LLHKPIVWHVNNATN	HLA DRB1*15:01, HLA DRB1*15:02	-0.036794254
	1986	LHKPIVWHVNNATNK	HLA DRB1*15:01, HLA DRB1*15:02, HLA DRB1*16:02	-0.19683503
	1987	HKPIVWHVNNATNKA	HLA DRB1*15:01, HLA DRB1*15:02, HLA DRB1*16:02	-0.2423791
	1988	KPIVWHVNNATNKAT	HLA DRB1*15:01, HLA DRB1*15:02	-0.19759012
	2015	STKPVETSNSFDVLK	HLA DRB1*07:01	-0.54491285
	2016	TKPVETSNSFDVLKS	HLA DRB1*07:01	-0.66171762
	2020	ETSNSFDVLKSEDAQ	HLA DRB1*16:02	-0.30753224
	2021	TSNSFDVLKSEDAQG	HLA DRB1*16:02	-0.070466066
	2022	SNSFDVLKSEDAQGM	HLA DRB1*16:02	-0.055015627
	2023	NSFDVLKSEDAQGMD	HLA DRB1*16:02	-0.36207716
	2096	MAAYVDNSSLTIKKP	HLA DRB1*07:01, HLA DRB1*16:02	-0.31825308

	2114	SRVLGLKTLATHGLA	HLA DRB1*12:02	**0.51038277**
	2115	RVLGLKTLATHGLAA	HLA DRB1*12:02	**0.43266595**
	2116	VLGLKTLATHGLAAV	HLA DRB1*12:02	**0.323982**
	2134	PWDTIANYAKPFLNK	HLA DRB1*15:01	-0.25570219
	2135	WDTIANYAKPFLNKV	HLA DRB1*15:01	-0.54848052
	2144	PFLNKVVSTTTNIVT	HLA DRB1*07:01	-0.29998765
	2145	FLNKVVSTTTNIVTR	HLA DRB1*07:01	-0.4301579
	2146	LNKVVSTTTNIVTRC	HLA DRB1*07:01	-0.3782325
	2147	NKVVSTTTNIVTRCL	HLA DRB1*07:01	-0.39253427
	2178	QLCTFTRSTNSRIKA	HLA DRB1*07:01	-0.21777362
	2179	LCTFTRSTNSRIKAS	HLA DRB1*07:01	-0.095922436
	2185	STNSRIKASMPTTIA	HLA DRB1*07:01	-0.49616959
	2186	TNSRIKASMPTTIAK	HLA DRB1*07:01	-0.18805364
	2187	NSRIKASMPTTIAKN	HLA DRB1*07:01	-0.42536127
	2210	CLEASFNYLKSPNFS	HLA DRB1*07:01, HLA DRB1*12:02, HLA DRB1*16:02	-0.094357236
	2211	LEASFNYLKSPNFSK	HLA DRB1*07:01, HLA DRB1*12:02, HLA DRB1*15:02, HLA DRB1*16:02	-0.12722255
	2212	EASFNYLKSPNFSKL	HLA DRB1*07:01, HLA DRB1*12:02, HLA DRB1*15:02, HLA DRB1*16:02	-0.37773341
	2213	ASFNYLKSPNFSKLI	HLA DRB1*16:02	-0.026528299
	2238	VCLGSLIYSTAALGV	HLA DRB1*07:01	**0.49861583**
	2239	CLGSLIYSTAALGVL	HLA DRB1*07:01	**0.35282643**
	2240	LGSLIYSTAALGVLM	HLA DRB1*07:01	**0.49521512**
	2241	GSLIYSTAALGVLMS	HLA DRB1*07:01	**0.30884964**
	2264	TGYREGYLNSTNVTI	HLA DRB1*07:01	-0.41173481
	2265	GYREGYLNSTNVTIA	HLA DRB1*07:01	-0.24449529
	2266	YREGYLNSTNVTIAT	HLA DRB1*07:01, HLA DRB1*16:02	-0.43067608
	2267	REGYLNSTNVTIATY	HLA DRB1*07:01, HLA DRB1*15:02, HLA DRB1*16:02	-0.31028204
	2268	EGYLNSTNVTIATYC	HLA DRB1*07:01	-0.33203241
	2294	GLDSLDTYPSLETIQ	HLA DRB1*15:01, HLA DRB1*15:02	-0.34549467
	2295	LDSLDTYPSLETIQI	HLA DRB1*15:01, HLA DRB1*15:02	-0.54310262
	2296	DSLDTYPSLETIQIT	HLA DRB1*15:01	-0.19638859
	2387	FASFYYVWKSYVHVV	HLA DRB1*15:02	**0.39201913**
	2388	ASFYYVWKSYVHVVD	HLA DRB1*15:02, HLA DRB1*16:02	**0.15218183**
	2429	GVRRSFYVYANGGKG	HLA DRB1*15:02	-0.32869375
	2430	VRRSFYVYANGGKGF	HLA DRB1*15:02, HLA DRB1*16:02	-0.50732704
	2431	RRSFYVYANGGKGFC	HLA DRB1*16:02	-0.20644744
	2496	VKNGSIHLYFDKAGQ	HLA DRB1*15:01, HLA DRB1*15:02	-0.69338588
	2497	KNGSIHLYFDKAGQK	HLA DRB1*15:01, HLA DRB1*15:02	-0.60597528
	2498	NGSIHLYFDKAGQKT	HLA DRB1*15:01, HLA DRB1*15:02	-0.53246273

	2499	GSIHLYFDKAGQKTY	HLA DRB1*15:01, HLA DRB1*15:02	-0.35732381
	2516	HSLSHFVNLDNLRAN	HLA DRB1*16:02	-0.22981627
	2517	SLSHFVNLDNLRANN	HLA DRB1*11:01, HLA DRB1*15:02, HLA DRB1*16:02	-0.17816212
	2518	LSHFVNLDNLRANNT	HLA DRB1*11:01, HLA DRB1*12:02, HLA DRB1*15:02, HLA DRB1*16:02	-0.29350215
	2519	SHFVNLDNLRANNTK	HLA DRB1*11:01, HLA DRB1*16:02	-0.40477542
	2585	EVAVKMFDAYVNTFS	HLA DRB1*15:01	-0.25130725
	2587	AVKMFDAYVNTFSST	HLA DRB1*15:02	-0.32388736
	2588	VKMFDAYVNTFSSTF	HLA DRB1*15:02	-0.42142796
	2590	MFDAYVNTFSSTFNV	HLA DRB1*07:01	-0.41134871
	2591	FDAYVNTFSSTFNVP	HLA DRB1*07:01	-0.56557317
	2592	DAYVNTFSSTFNVPM	HLA DRB1*07:01	-0.50623878
	2593	AYVNTFSSTFNVPME	HLA DRB1*07:01	-0.20604344
	2594	YVNTFSSTFNVPMEK	HLA DRB1*07:01	-0.12732785
	2595	VNTFSSTFNVPMEKL	HLA DRB1*07:01	-0.18682741
	2602	FNVPMEKLKTLVATA	HLA DRB1*12:02	**0.16860267**
	2603	NVPMEKLKTLVATAE	HLA DRB1*12:02	**0.32043407**
	2608	KLKTLVATAEAELAK	HLA DRB1*07:01	**0.28758531**
	2609	LKTLVATAEAELAKN	HLA DRB1*07:01	**0.28217656**
	2626	LDNVLSTFISAARQG	HLA DRB1*16:02	**0.25499401**
	2627	DNVLSTFISAARQGF	HLA DRB1*16:02	**0.060853447**
	2721	LSEQLRKQIRSAAKK	HLA DRB1*11:01	-0.43971802
	2722	SEQLRKQIRSAAKKN	HLA DRB1*11:01	-0.37653178
	2725	LRKQIRSAAKKNNLP	HLA DRB1*11:01	-0.59829081
	2726	RKQIRSAAKKNNLPF	HLA DRB1*11:01	-0.40678367
	2745	ATTRQVVNVVTTKIA	HLA DRB1*07:01	-0.31787192
	2747	TRQVVNVVTTKIALK	HLA DRB1*07:01	**0.02810012**
	2782	FVAAIFYLITPVHVM	HLA DRB1*07:01	-0.27424945
	2783	VAAIFYLITPVHVMS	HLA DRB1*07:01, HLA DRB1*16:02	-0.089830143
	2784	AAIFYLITPVHVMSK	HLA DRB1*07:01, HLA DRB1*16:02	-0.54022313
	2785	AIFYLITPVHVMSKH	HLA DRB1*07:01	-0.76771484
	2789	LITPVHVMSKHTDFS	HLA DRB1*11:01	-0.04426137
	2789	LITPVHVMSKHTDFS	HLA DRB1*12:02	-0.13378443
	2790	ITPVHVMSKHTDFSS	HLA DRB1*11:01, HLA DRB1*12:02	-0.39904248
	2800	TDFSSEIIGYKAIDG	HLA DRB1*15:01	-0.27549762
	2801	DFSSEIIGYKAIDGG	HLA DRB1*15:01, HLA DRB1*15:02, HLA DRB1*16:02	-0.51805862
	2802	FSSEIIGYKAIDGGV	HLA DRB1*15:01, HLA DRB1*15:02, HLA DRB1*16:02	-0.78718709
	2803	SSEIIGYKAIDGGVT	HLA DRB1*15:01, HLA DRB1*15:02, HLA DRB1*16:02	-0.5932268
	2804	SEIIGYKAIDGGVTR	HLA DRB1*15:01, HLA DRB1*15:02, HLA DRB1*16:02	-0.56631424
	2805	EIIGYKAIDGGVTRD	HLA DRB1*15:01, HLA DRB1*16:02	-0.72997846

	2806	IIGYKAIDGGVTRDI	HLA DRB1*16:02	**0.27999202**
	2854	IAAVITREVGFVVPG	HLA DRB1*15:02	-0.0012576928
	2877	TNGDFLHFLPRVFSA	HLA DRB1*11:01	**0.11037918**
	2878	NGDFLHFLPRVFSAV	HLA DRB1*11:01	**0.31918441**
	2879	GDFLHFLPRVFSAVG	HLA DRB1*11:01	**0.58060762**
	2890	SAVGNICYTPSKLIE	HLA DRB1*07:01	**0.51851408**
	2891	AVGNICYTPSKLIEY	HLA DRB1*07:01	**0.31850723**
	2892	VGNICYTPSKLIEYT	HLA DRB1*07:01	**0.36940461**
	2893	GNICYTPSKLIEYTD	HLA DRB1*07:01	**0.17370758**
	2897	YTPSKLIEYTDFATS	HLA DRB1*15:01, HLA DRB1*15:02	**0.20829641**
	2898	TPSKLIEYTDFATSA	HLA DRB1*15:01, HLA DRB1*15:02	**0.4148902**
	2899	PSKLIEYTDFATSAC	HLA DRB1*15:01, HLA DRB1*15:02	**0.17836648**
	2900	SKLIEYTDFATSACV	HLA DRB1*15:01	-0.7519447
	2918	ECTIFKDASGKPVPY	HLA DRB1*07:01	**0.0087233443**
	2949	RPDTRYVLMDGSIIQ	HLA DRB1*16:02	-0.53836862
	2950	PDTRYVLMDGSIIQF	HLA DRB1*15:02, HLA DRB1*16:02	-0.4605804
	2951	DTRYVLMDGSIIQFP	HLA DRB1*15:02, HLA DRB1*16:02	-0.07450474
	2952	TRYVLMDGSIIQFPN	HLA DRB1*16:02	-0.4932638
	2955	VLMDGSIIQFPNTYL	HLA DRB1*15:01, HLA DRB1*15:02	-0.33435363
	2956	LMDGSIIQFPNTYLE	HLA DRB1*15:01, HLA DRB1*15:02, HLA DRB1*16:02	-0.25075639
	2957	MDGSIIQFPNTYLEG	HLA DRB1*15:01, HLA DRB1*15:02, HLA DRB1*16:02	**0.21025374**
	2958	DGSIIQFPNTYLEGS	HLA DRB1*12:02, HLA DRB1*15:01, HLA DRB1*15:02, HLA DRB1*16:02	**0.087720744**
	2959	GSIIQFPNTYLEGSV	HLA DRB1*15:01, HLA DRB1*15:02, HLA DRB1*16:02	**0.25912222**
	2960	SIIQFPNTYLEGSVR	HLA DRB1*15:01, HLA DRB1*15:02	-0.78467881
	2972	SVRVVTTFDSEYCRH	HLA DRB1*15:01	-0.37523635
	2973	VRVVTTFDSEYCRHG	HLA DRB1*15:01, HLA DRB1*15:02	-0.066934048
	3006	LNNDYYRSLPGVFCG	HLA DRB1*16:02	**0.39976925**
	3007	NNDYYRSLPGVFCGV	HLA DRB1*16:02	**0.32931549**
	3008	NDYYRSLPGVFCGVD	HLA DRB1*16:02	-0.41015989
	3028	TNMFTPLIQPIGALD	HLA DRB1*11:01	-0.065329836
	3030	MFTPLIQPIGALDIS	HLA DRB1*07:01	**0.15814274**
	3031	FTPLIQPIGALDISA	HLA DRB1*07:01, HLA DRB1*12:02	**0.20744102**
	3032	TPLIQPIGALDISAS	HLA DRB1*07:01, HLA DRB1*12:02	-0.086129717
	3060	LAYYFMRFRRAFGEY	HLA DRB1*11:01	-0.2289568
	3061	AYYFMRFRRAFGEYS	HLA DRB1*11:01	**0.58437182**
	3094	LTPVYSFLPGVYSVI	HLA DRB1*16:02	**0.56612589**
	3095	TPVYSFLPGVYSVIY	HLA DRB1*16:02	-0.046689717
	3105	YSVIYLYLTFYLTND	HLA DRB1*15:01	-0.17658548

	3148	ISTKHFYWFFSNYLK	HLA DRB1*15:02, HLA DRB1*16:02	-0.34242601
	3149	STKHFYWFFSNYLKR	HLA DRB1*15:01, HLA DRB1*15:02, HLA DRB1*16:02	-0.45241268
	3150	TKHFYWFFSNYLKRR	HLA DRB1*07:01, HLA DRB1*15:01, HLA DRB1*15:02, HLA DRB1*16:02	-0.34583424
	3151	KHFYWFFSNYLKRRV	HLA DRB1*15:01, HLA DRB1*15:02, HLA DRB1*16:02	-0.11907884
	3209	ALYNKYKYFSGAMDT	HLA DRB1*16:02	-0.26436815
	3210	LYNKYKYFSGAMDTT	HLA DRB1*15:02, HLA DRB1*16:02	-0.49044619
	3211	YNKYKYFSGAMDTTS	HLA DRB1*15:02, HLA DRB1*16:02	-0.40499908
	3212	NKYKYFSGAMDTTSY	HLA DRB1*16:02	**0.034925467**
	3326	NHNFLVQAGNVQLRV	HLA DRB1*15:02, HLA DRB1*16:02	-0.22907385
	3359	PKTPKYKFVRIQPGQ	HLA DRB1*07:01, HLA DRB1*11:01	-0.26182997
	3360	KTPKYKFVRIQPGQT	HLA DRB1*07:01, HLA DRB1*11:01	-0.42327254
	3361	TPKYKFVRIQPGQTF	HLA DRB1*07:01, HLA DRB1*11:01	-0.17899644
	3362	PKYKFVRIQPGQTFS	HLA DRB1*11:01	-0.037425607
	3363	KYKFVRIQPGQTFSV	HLA DRB1*11:01	**0.16151551**
	3564	SGVTFQSAVKRTIKG	HLA DRB1*11:01	**0.1891356**
	3565	GVTFQSAVKRTIKGT	HLA DRB1*11:01	**0.16802027**
	3713	NVLTLVYKVYYGNAL	HLA DRB1*15:02	**0.17334471**
	3714	VLTLVYKVYYGNALD	HLA DRB1*15:01, HLA DRB1*15:02, HLA DRB1*16:02	**0.17563228**
	3715	LTLVYKVYYGNALDQ	HLA DRB1*15:01, HLA DRB1*15:02, HLA DRB1*16:02	-0.12812212
	3716	TLVYKVYYGNALDQA	HLA DRB1*15:01, HLA DRB1*15:02, HLA DRB1*16:02	**0.0070755559**
	3717	LVYKVYYGNALDQAI	HLA DRB1*15:01, HLA DRB1*15:02, HLA DRB1*16:02	-0.019808209
	3718	VYKVYYGNALDQAIS	HLA DRB1*15:02	**0.17021009**
	3814	LVSTQEFRYMNSQGL	HLA DRB1*07:01, HLA DRB1*15:02, HLA DRB1*16:02	-0.039172088
	3815	VSTQEFRYMNSQGLL	HLA DRB1*07:01, HLA DRB1*15:02, HLA DRB1*16:02	**0.097632046**
	3816	STQEFRYMNSQGLLP	HLA DRB1*07:01, HLA DRB1*12:02, HLA DRB1*15:01, HLA DRB1*15:02, HLA DRB1*16:02	-0.28198599
	3817	TQEFRYMNSQGLLPP	HLA DRB1*07:01, HLA DRB1*12:02, HLA DRB1*15:01, HLA DRB1*15:02, HLA DRB1*16:02	-0.41760878
	3818	QEFRYMNSQGLLPPK	HLA DRB1*07:01, HLA DRB1*12:02, HLA DRB1*15:01, HLA DRB1*15:02, HLA DRB1*16:02	-0.25437902
	3819	EFRYMNSQGLLPPKN	HLA DRB1*16:02	-0.062325272
	3833	NSIDAFKLNIKLLGV	HLA DRB1*11:01	**0.34258395**
	3834	SIDAFKLNIKLLGVG	HLA DRB1*11:01	**0.23506756**
	3835	IDAFKLNIKLLGVGG	HLA DRB1*11:01	-0.10531173
	3836	DAFKLNIKLLGVGGK	HLA DRB1*11:01	**0.49673838**
	3943	AIASEFSSLPSYAAF	HLA DRB1*16:02	-0.082021259
	3944	IASEFSSLPSYAAFA	HLA DRB1*15:02, HLA DRB1*16:02	**0.075448812**
	3945	ASEFSSLPSYAAFAT	HLA DRB1*15:02, HLA DRB1*16:02	**0.3972671**
	3946	SEFSSLPSYAAFATA	HLA DRB1*16:02	**0.2211394**
	3950	SLPSYAAFATAQEAY	HLA DRB1*16:02	**0.17363263**

	3951	LPSYAAFATAQEAYE	HLA DRB1*16:02	**0.05175581**
	3959	TAQEAYEQAVANGDS	HLA DRB1*07:01	-0.15963941
	3960	AQEAYEQAVANGDSE	HLA DRB1*07:01	-0.46428452
	3961	QEAYEQAVANGDSEV	HLA DRB1*07:01	-0.54067076
	3974	EVVLKKLKKSLNVAK	HLA DRB1*11:01	**0.6329412**
	4029	MQTMLFTMLRKLDND	HLA DRB1*11:01	-0.20734393
	4030	QTMLFTMLRKLDNDA	HLA DRB1*11:01	-0.087059221
	4031	TMLFTMLRKLDNDAL	HLA DRB1*11:01	-0.04278137
	4068	AKLMVVIPDYNTYKN	HLA DRB1*15:01	-0.42757332
	4103	DADSKIVQLSEISMD	HLA DRB1*12:02	-0.61372399
	4104	ADSKIVQLSEISMDN	HLA DRB1*12:02	-0.76106239
	4105	DSKIVQLSEISMDNS	HLA DRB1*12:02	-0.69078384
	4106	SKIVQLSEISMDNSP	HLA DRB1*12:02	-1.1295165
	4124	WPLIVTALRANSAVK	HLA DRB1*11:01, HLA DRB1*12:02	**0.78569358**
	4125	PLIVTALRANSAVKL	HLA DRB1*12:02	**0.63818085**
	4128	VTALRANSAVKLQNN	HLA DRB1*07:01	**0.41017385**
	4164	TDDNALAYYNTTKGG	HLA DRB1*15:01, HLA DRB1*15:02	-0.61761105
	4165	DDNALAYYNTTKGGR	HLA DRB1*15:01, HLA DRB1*15:02, HLA DRB1*16:02	-0.42845902
	4166	DNALAYYNTTKGGRF	HLA DRB1*15:01, HLA DRB1*15:02, HLA DRB1*16:02	-0.74369435
	4167	NALAYYNTTKGGRFV	HLA DRB1*07:01, HLA DRB1*15:01, HLA DRB1*15:02	-0.4713522
	4168	ALAYYNTTKGGRFVL	HLA DRB1*07:01	-0.47916381
	4169	LAYYNTTKGGRFVLA	HLA DRB1*07:01	-0.057237948
	4189	QDLKWARFPKSDGTG	HLA DRB1*11:01	-0.65990954
	4190	DLKWARFPKSDGTGT	HLA DRB1*11:01	-0.78591897
	4222	GPKVKYLYFIKGLNN	HLA DRB1*11:01	-0.16032734
	4223	PKVKYLYFIKGLNNL	HLA DRB1*11:01	**0.16674258**
	4224	KVKYLYFIKGLNNLN	HLA DRB1*11:01	-0.0030323981
	4242	VLGSLAATVRLQAGN	HLA DRB1*11:01	**0.12826589**
	4243	LGSLAATVRLQAGNA	HLA DRB1*11:01	**0.10055086**
	4244	GSLAATVRLQAGNAT	HLA DRB1*11:01	**0.064312226**
	4262	ANSTVLSFCAFAVDA	HLA DRB1*15:01	-0.0537201
	4263	NSTVLSFCAFAVDAA	HLA DRB1*15:01, HLA DRB1*15:02	**0.048452802**
	4272	FAVDAAKAYKDYLAS	HLA DRB1*15:01	**0.3387717**
	4273	AVDAAKAYKDYLASG	HLA DRB1*15:01	**0.22276241**
	4274	VDAAKAYKDYLASGG	HLA DRB1*15:01, HLA DRB1*15:02	-0.11036117
	4275	DAAKAYKDYLASGGQ	HLA DRB1*15:01	-0.13857939
	4276	AAKAYKDYLASGGQP	HLA DRB1*15:02	-0.050270034
	4277	AKAYKDYLASGGQPI	HLA DRB1*07:01, HLA DRB1*15:02	**0.27241329**
	4278	KAYKDYLASGGQPIT	HLA DRB1*07:01, HLA DRB1*16:02	**0.19650355**
	4279	AYKDYLASGGQPITN	HLA DRB1*07:01, HLA DRB1*15:02, HLA DRB1*16:02	**0.01567761**

	4280	YKDYLASGGQPITNC	HLA DRB1*07:01, HLA DRB1*16:02	**0.14080429**
	4281	KDYLASGGQPITNCV	HLA DRB1*07:01	**0.14655744**
	4345	LKGKYVQIPTTCAND	HLA DRB1*11:01, HLA DRB1*16:02	-0.22354251
	4346	KGKYVQIPTTCANDP	HLA DRB1*11:01, HLA DRB1*16:02	-0.3406529
	4418	TSTDVVYRAFDIYND	HLA DRB1*15:01, HLA DRB1*15:02	**0.041567617**
	4422	VVYRAFDIYNDKVAG	HLA DRB1*15:01, HLA DRB1*15:02	**0.072694755**
	4423	VYRAFDIYNDKVAGF	HLA DRB1*15:01, HLA DRB1*15:02	-0.12274593
	4424	YRAFDIYNDKVAGFA	HLA DRB1*15:01, HLA DRB1*15:02, HLA DRB1*16:02	**0.089073011**
	4425	RAFDIYNDKVAGFAK	HLA DRB1*15:01, HLA DRB1*15:02	**0.018452115**
	4456	NLIDSYFVVKRHTFS	HLA DRB1*11:01	**0.19160503**
	4457	LIDSYFVVKRHTFSN	HLA DRB1*11:01, HLA DRB1*12:02	**0.13041491**
	4458	IDSYFVVKRHTFSNY	HLA DRB1*11:01, HLA DRB1*12:02	**0.1870334**
	4459	DSYFVVKRHTFSNYQ	HLA DRB1*11:01	**0.17255265**
	4551	KKDWYDFVENPDILR	HLA DRB1*16:02	**0.077198278**
	4552	KDWYDFVENPDILRV	HLA DRB1*16:02	**0.10212444**
	4559	ENPDILRVYANLGER	HLA DRB1*12:02, HLA DRB1*15:01, HLA DRB1*15:02, HLA DRB1*16:02	**0.011725622**
	4560	NPDILRVYANLGERV	HLA DRB1*12:02, HLA DRB1*15:01, HLA DRB1*15:02, HLA DRB1*16:02	**0.22993566**
	4561	PDILRVYANLGERVR	HLA DRB1*12:02, HLA DRB1*15:01, HLA DRB1*15:02, HLA DRB1*16:02	**0.26161469**
	4562	DILRVYANLGERVRQ	HLA DRB1*15:01, HLA DRB1*15:02	**0.19512384**
	4610	DFGDFIQTTPGSGVP	HLA DRB1*07:01	-0.15132234
	4611	FGDFIQTTPGSGVPV	HLA DRB1*07:01	-0.18966827
	4612	GDFIQTTPGSGVPVV	HLA DRB1*07:01	-0.13007353
	4622	GVPVVDSYYSLLMPI	HLA DRB1*15:01	-0.054402262
	4634	MPILTLTRALTAESH	HLA DRB1*07:01	**0.28242312**
	4675	FDRYFKYWDQTYHPN	HLA DRB1*15:02	**0.24811396**
	4727	VDGVPFVVSTGYHFR	HLA DRB1*07:01, HLA DRB1*16:02	**0.11480612**
	4728	DGVPFVVSTGYHFRE	HLA DRB1*07:01, HLA DRB1*15:02, HLA DRB1*16:02	**0.03879744**
	4729	GVPFVVSTGYHFREL	HLA DRB1*07:01, HLA DRB1*15:02, HLA DRB1*16:02	**0.081607683**
	4730	VPFVVSTGYHFRELG	HLA DRB1*07:01	**0.23177467**
	4758	LSFKELLVYAADPAM	HLA DRB1*15:01, HLA DRB1*15:02	-0.077004067
	4759	SFKELLVYAADPAMH	HLA DRB1*15:01, HLA DRB1*15:02, HLA DRB1*16:02	-0.27908132
	4760	FKELLVYAADPAMHA	HLA DRB1*15:01, HLA DRB1*15:02, HLA DRB1*16:02	**0.11371587**
	4761	KELLVYAADPAMHAA	HLA DRB1*15:01, HLA DRB1*15:02, HLA DRB1*16:02	**0.22582922**
	4768	ADPAMHAASGNLLLD	HLA DRB1*07:01	-0.68330658
	4769	DPAMHAASGNLLLDK	HLA DRB1*07:01	-0.58368375
	4795	NNVAFQTVKPGNFNK	HLA DRB1*07:01	-0.11573911
	4796	NVAFQTVKPGNFNKD	HLA DRB1*07:01	-0.10606629

	4807	FNKDFYDFAVSKGFF	HLA DRB1*07:01	-0.40289788
	4808	NKDFYDFAVSKGFFK	HLA DRB1*07:01	-0.079483904
	4809	KDFYDFAVSKGFFKE	HLA DRB1*07:01	-0.14047237
	4817	SKGFFKEGSSVELKH	HLA DRB1*07:01	-0.67956316
	4818	KGFFKEGSSVELKHF	HLA DRB1*07:01	-0.52497763
	4829	LKHFFFAQDGNAAIS	HLA DRB1*16:02	**0.26791847**
	4830	KHFFFAQDGNAAISD	HLA DRB1*15:02, HLA DRB1*16:02	**0.44008259**
	4886	IVNNLDKSAGFPFNK	HLA DRB1*07:01	-0.40376859
	4915	DQDALFAYTKRNVIP	HLA DRB1*11:01	-0.3368985
	4916	QDALFAYTKRNVIPT	HLA DRB1*11:01	-0.060695981
	4917	DALFAYTKRNVIPTI	HLA DRB1*11:01	-0.26013623
	4932	TQMNLKYAISAKNRA	HLA DRB1*11:01	**0.35183292**
	4933	QMNLKYAISAKNRAR	HLA DRB1*11:01	**0.40443361**
	4934	MNLKYAISAKNRART	HLA DRB1*11:01	**0.40189009**
	4935	NLKYAISAKNRARTV	HLA DRB1*11:01	**0.59384444**
	4936	LKYAISAKNRARTVA	HLA DRB1*11:01	**0.65441232**
	4937	KYAISAKNRARTVAG	HLA DRB1*11:01	**0.58340402**
	4965	QKLLKSIAATRGATV	HLA DRB1*07:01	**0.2049577**
	4966	KLLKSIAATRGATVV	HLA DRB1*07:01	**0.40943023**
	4967	LLKSIAATRGATVVI	HLA DRB1*07:01	**0.39407314**
	4968	LKSIAATRGATVVIG	HLA DRB1*07:01	**0.51630525**
	4969	KSIAATRGATVVIGT	HLA DRB1*07:01	**0.62202121**
	5018	MPNMLRIMASLVLAR	HLA DRB1*12:02, HLA DRB1*15:01, HLA DRB1*15:02, HLA DRB1*16:02	**0.32014953**
	5019	PNMLRIMASLVLARK	HLA DRB1*12:02, HLA DRB1*15:01, HLA DRB1*15:02, HLA DRB1*16:02	**0.39136883**
	5020	NMLRIMASLVLARKH	HLA DRB1*15:01, HLA DRB1*15:02, HLA DRB1*16:02	**0.13368254**
	5093	TANVNALLSTDGNKI	HLA DRB1*07:01	-0.1135887
	5094	ANVNALLSTDGNKIA	HLA DRB1*07:01	-0.30390308
	5095	NVNALLSTDGNKIAD	HLA DRB1*07:01	-0.021819874
	5096	VNALLSTDGNKIADK	HLA DRB1*07:01, HLA DRB1*15:02, HLA DRB1*16:02	**0.22113402**
	5097	NALLSTDGNKIADKY	HLA DRB1*07:01	**0.20149438**
	5106	KIADKYVRNLQHRLY	HLA DRB1*12:02	-0.23107785
	5107	IADKYVRNLQHRLYE	HLA DRB1*12:02	-0.31784011
	5108	ADKYVRNLQHRLYEC	HLA DRB1*12:02	-0.30902884
	5109	DKYVRNLQHRLYECL	HLA DRB1*12:02	-0.27625912
	5115	LQHRLYECLYRNRDV	HLA DRB1*11:01	-0.18997364
	5116	QHRLYECLYRNRDVD	HLA DRB1*11:01	-0.11496807
	5117	HRLYECLYRNRDVDT	HLA DRB1*11:01	-0.14108854
	5118	RLYECLYRNRDVDTD	HLA DRB1*11:01	-0.30582316

	5132	DFVNEFYAYLRKHFS	HLA DRB1*11:01	-0.20027092
	5133	FVNEFYAYLRKHFSM	HLA DRB1*11:01	-0.63092501
	5134	VNEFYAYLRKHFSMM	HLA DRB1*11:01	-0.52175242
	5135	NEFYAYLRKHFSMMI	HLA DRB1*11:01	-0.26378918
	5136	EFYAYLRKHFSMMIL	HLA DRB1*11:01	-0.23100353
	5150	LSDDAVVCFNSTYAS	HLA DRB1*15:01, HLA DRB1*15:02	**0.034561966**
	5151	SDDAVVCFNSTYASQ	HLA DRB1*15:01, HLA DRB1*15:02, HLA DRB1*16:02	**0.026386468**
	5152	DDAVVCFNSTYASQG	HLA DRB1*15:01, HLA DRB1*15:02, HLA DRB1*16:02	-0.088754083
	5153	DAVVCFNSTYASQGL	HLA DRB1*15:01	**0.33611739**
	5164	SQGLVASIKNFKSVL	HLA DRB1*15:01	**0.37231794**
	5165	QGLVASIKNFKSVLY	HLA DRB1*12:02, HLA DRB1*15:01	**0.24848213**
	5166	GLVASIKNFKSVLYY	HLA DRB1*12:02, HLA DRB1*15:01, HLA DRB1*15:02, HLA DRB1*16:02	**0.43106149**
	5167	LVASIKNFKSVLYYQ	HLA DRB1*12:02, HLA DRB1*15:01, HLA DRB1*15:02, HLA DRB1*16:02	**0.093707023**
	5168	VASIKNFKSVLYYQN	HLA DRB1*12:02, HLA DRB1*15:01, HLA DRB1*15:02, HLA DRB1*16:02	**0.29825337**
	5169	ASIKNFKSVLYYQNN	HLA DRB1*12:02, HLA DRB1*15:01, HLA DRB1*15:02, HLA DRB1*16:02	**0.25775961**
	5172	KNFKSVLYYQNNVFM	HLA DRB1*15:01, HLA DRB1*15:02	-0.014358134
	5173	NFKSVLYYQNNVFMS	HLA DRB1*15:01, HLA DRB1*15:02	**0.096870191**
	5174	FKSVLYYQNNVFMSE	HLA DRB1*15:01, HLA DRB1*15:02, HLA DRB1*16:02	-0.00094937127
	5175	KSVLYYQNNVFMSEA	HLA DRB1*15:01, HLA DRB1*15:02, HLA DRB1*16:02	**0.13527738**
	5234	CFVDDIVKTDGTLMI	HLA DRB1*07:01	-0.88380089
	5235	FVDDIVKTDGTLMIE	HLA DRB1*07:01	-0.90124302
	5236	VDDIVKTDGTLMIER	HLA DRB1*07:01	-0.84103604
	5237	DDIVKTDGTLMIERF	HLA DRB1*07:01	-1.1896573
	5244	GTLMIERFVSLAIDA	HLA DRB1*15:01, HLA DRB1*15:02	-0.82427027
	5245	TLMIERFVSLAIDAY	HLA DRB1*15:01, HLA DRB1*15:02	-0.65076675
	5362	SHKLVLSVNPYVCNA	HLA DRB1*15:02	-0.35281523
	5410	NGQVFGLYKNTCVGS	HLA DRB1*15:02	-0.2834386
	5411	GQVFGLYKNTCVGSD	HLA DRB1*15:01, HLA DRB1*15:02	**0.12639966**
	5449	TCTERLKLFAAETLK	HLA DRB1*15:01, HLA DRB1*15:02, HLA DRB1*16:02	-0.27901465
	5450	CTERLKLFAAETLKA	HLA DRB1*12:02, HLA DRB1*15:01, HLA DRB1*15:02, HLA DRB1*16:02	-0.10920914
	5451	TERLKLFAAETLKAT	HLA DRB1*07:01, HLA DRB1*12:02, HLA DRB1*15:01, HLA DRB1*15:02, HLA DRB1*16:02	**0.013340883**
	5452	ERLKLFAAETLKATE	HLA DRB1*15:01, HLA DRB1*15:02, HLA DRB1*16:02	**0.027634477**
	5465	TEETFKLSYGIATVR	HLA DRB1*07:01	-0.15300691
	5466	EETFKLSYGIATVRE	HLA DRB1*07:01	-0.13727134
	5470	KLSYGIATVREVLSD	HLA DRB1*07:01	**0.043163927**

	5503	NYVFTGYRVTKNSKV	HLA DRB1*07:01	-0.78894867
	5504	YVFTGYRVTKNSKVQ	HLA DRB1*07:01, HLA DRB1*15:02, HLA DRB1*16:02	-0.76041811
	5505	VFTGYRVTKNSKVQI	HLA DRB1*07:01, HLA DRB1*15:02, HLA DRB1*16:02	-0.70114021
	5506	FTGYRVTKNSKVQIG	HLA DRB1*07:01, HLA DRB1*15:02, HLA DRB1*16:02	-0.7629068
	5507	TGYRVTKNSKVQIGE	HLA DRB1*07:01, HLA DRB1*15:02, HLA DRB1*16:02	-0.81396755
	5508	GYRVTKNSKVQIGEY	HLA DRB1*07:01	-0.53448585
	5529	YGDAVVYRGTTTYKL	HLA DRB1*07:01	-0.36532217
	5530	GDAVVYRGTTTYKLN	HLA DRB1*07:01	-0.36697071
	5531	DAVVYRGTTTYKLNV	HLA DRB1*07:01, HLA DRB1*16:02	-0.0019186367
	5532	AVVYRGTTTYKLNVG	HLA DRB1*07:01, HLA DRB1*15:02, HLA DRB1*16:02	-0.021410949
	5533	VVYRGTTTYKLNVGD	HLA DRB1*07:01	-0.1363368
	5534	VYRGTTTYKLNVGDY	HLA DRB1*07:01	-0.16206678
	5542	KLNVGDYFVLTSHTV	HLA DRB1*16:02	-0.15056949
	5543	LNVGDYFVLTSHTVM	HLA DRB1*15:02, HLA DRB1*16:02	-0.36042586
	5544	NVGDYFVLTSHTVMP	HLA DRB1*07:01, HLA DRB1*15:02, HLA DRB1*16:02	-0.43765126
	5545	VGDYFVLTSHTVMPL	HLA DRB1*07:01, HLA DRB1*15:02, HLA DRB1*16:02	-0.17329827
	5546	GDYFVLTSHTVMPLS	HLA DRB1*15:02, HLA DRB1*16:02	-0.31357192
	5553	SHTVMPLSAPTLVPQ	HLA DRB1*12:02, HLA DRB1*15:01	-0.54038934
	5594	QKVGMQKYSTLQGPP	HLA DRB1*15:01, HLA DRB1*15:02	-0.81414195
	5595	KVGMQKYSTLQGPPG	HLA DRB1*15:01, HLA DRB1*15:02	-0.67029702
	5596	VGMQKYSTLQGPPGT	HLA DRB1*15:01	-0.73912984
	5598	MQKYSTLQGPPGTGK	HLA DRB1*16:02	-0.64959807
	5616	AIGLALYYPSARIVY	HLA DRB1*15:01	**0.20585782**
	5617	IGLALYYPSARIVYT	HLA DRB1*15:01	**0.18608175**
	5666	CFDKFKVNSTLEQYV	HLA DRB1*11:01	-0.093789713
	5667	FDKFKVNSTLEQYVF	HLA DRB1*11:01	-0.24532673
	5690	TTADIVVFDEISMAT	HLA DRB1*15:01	-0.41762994
	5691	TADIVVFDEISMATN	HLA DRB1*15:01, HLA DRB1*15:02	-0.42912485
	5696	VFDEISMATNYDLSV	HLA DRB1*15:01, HLA DRB1*15:02	-0.59626637
	5697	FDEISMATNYDLSVV	HLA DRB1*15:01, HLA DRB1*15:02	-0.50310201
	5716	RAKHYVYIGDPAQLP	HLA DRB1*16:02	-0.18755018
	5717	AKHYVYIGDPAQLPA	HLA DRB1*11:01, HLA DRB1*16:02	**0.16727452**
	5718	KHYVYIGDPAQLPAP	HLA DRB1*16:02	-0.3181297
	5767	RCPAEIVDTVSALVY	HLA DRB1*07:01	-0.20882629
	5768	CPAEIVDTVSALVYD	HLA DRB1*07:01, HLA DRB1*15:02	-0.24004956
	5769	PAEIVDTVSALVYDN	HLA DRB1*07:01, HLA DRB1*15:01, HLA DRB1*15:02, HLA DRB1*16:02	-0.31287444
	5770	AEIVDTVSALVYDNK	HLA DRB1*07:01, HLA DRB1*12:02	-0.46019668
	5774	DTVSALVYDNKLKAH	HLA DRB1*11:01	-0.044191882
	5775	TVSALVYDNKLKAHK	HLA DRB1*11:01	**0.35165561**

	5776	VSALVYDNKLKAHKD	HLA DRB1*11:01	**0.25601688**
	5777	SALVYDNKLKAHKDK	HLA DRB1*11:01	**0.49102369**
	5795	CFKMFYKGVITHDVS	HLA DRB1*07:01	-0.59974749
	5796	FKMFYKGVITHDVSS	HLA DRB1*07:01	-0.49172757
	5797	KMFYKGVITHDVSSA	HLA DRB1*07:01, HLA DRB1*16:02	-0.45539364
	5814	RPQIGVVREFLTRNP	HLA DRB1*12:02	-0.48461916
	5830	WRKAVFISPYNSQNA	HLA DRB1*15:01	**0.23383292**
	5831	RKAVFISPYNSQNAV	HLA DRB1*15:01, HLA DRB1*15:02	**0.53933214**
	5832	KAVFISPYNSQNAVA	HLA DRB1*15:01, HLA DRB1*15:02, HLA DRB1*16:02	**0.4720961**
	5833	AVFISPYNSQNAVAS	HLA DRB1*15:01, HLA DRB1*15:02, HLA DRB1*16:02	**0.53704543**
	5834	VFISPYNSQNAVASK	HLA DRB1*15:01, HLA DRB1*15:02, HLA DRB1*16:02	**0.22363359**
	5835	FISPYNSQNAVASKI	HLA DRB1*15:01	**0.043858555**
	5844	AVASKILGLPTQTVD	HLA DRB1*12:02	-0.07832972
	5845	VASKILGLPTQTVDS	HLA DRB1*12:02, HLA DRB1*15:01, HLA DRB1*15:02, HLA DRB1*16:02	**0.015678357**
	5846	ASKILGLPTQTVDSS	HLA DRB1*12:02, HLA DRB1*15:01, HLA DRB1*15:02, HLA DRB1*16:02	-0.12146879
	5847	SKILGLPTQTVDSSQ	HLA DRB1*12:02	-0.097107987
	5916	IPRRNVATLQAENVT	HLA DRB1*12:02	-0.11088735
	5917	PRRNVATLQAENVTG	HLA DRB1*12:02	**0.0014152941**
	5918	RRNVATLQAENVTGL	HLA DRB1*12:02	**0.22897269**
	5986	KMNYQVNGYPNMFIT	HLA DRB1*15:01, HLA DRB1*15:02	-0.32066822
	5987	MNYQVNGYPNMFITR	HLA DRB1*15:01, HLA DRB1*15:02	-0.1812924
	5988	NYQVNGYPNMFITRE	HLA DRB1*15:01, HLA DRB1*15:02, HLA DRB1*16:02	-0.22008393
	5989	YQVNGYPNMFITREE	HLA DRB1*15:01, HLA DRB1*15:02	-0.1390682
	6000	TREEAIRHVRAWIGF	HLA DRB1*12:02	**0.17190051**
	6001	REEAIRHVRAWIGFD	HLA DRB1*12:02, HLA DRB1*15:01, HLA DRB1*15:02	**0.11715273**
	6002	EEAIRHVRAWIGFDV	HLA DRB1*12:02, HLA DRB1*15:01, HLA DRB1*15:02 HLA DRB1*16:02	**0.42312165**
	6003	EAIRHVRAWIGFDVE	HLA DRB1*15:01, HLA DRB1*15:02	**0.23743019**
	6004	AIRHVRAWIGFDVEG	HLA DRB1*15:01, HLA DRB1*15:02	**0.2893765**
	6005	IRHVRAWIGFDVEGC	HLA DRB1*15:01, HLA DRB1*15:02	**0.59401836**
	6006	RHVRAWIGFDVEGCH	HLA DRB1*15:01	**0.18377954**
	6038	TGVNLVAVPTGYVDT	HLA DRB1*15:01, HLA DRB1*15:02, HLA DRB1*16:02	-0.25176918
	6039	GVNLVAVPTGYVDTP	HLA DRB1*07:01, HLA DRB1*12:02, HLA DRB1*15:01, HLA DRB1*15:02, HLA DRB1*16:02	-0.46784679
	6055	NTDFSRVSAKPPPGD	HLA DRB1*07:01	-0.23055851
	6066	PPGDQFKHLIPLMYK	HLA DRB1*12:02	-0.79302192
	6067	PGDQFKHLIPLMYKG	HLA DRB1*12:02	-0.66712494
	6068	GDQFKHLIPLMYKGL	HLA DRB1*12:02	-0.50207002
	6069	DQFKHLIPLMYKGLP	HLA DRB1*12:02	-0.77532188

	6087	VRIKIVQMLSDTLKN	HLA DRB1*12:02, HLA DRB1*15:01, HLA DRB1*15:02, HLA DRB1*16:02	-0.08846654
	6260	PKAIKCVPQADVEWK	HLA DRB1*12:02	**0.11520704**
	6268	QADVEWKFYDAQPCS	HLA DRB1*15:02, HLA DRB1*16:02	**0.093446735**
	6269	ADVEWKFYDAQPCSD	HLA DRB1*15:01, HLA DRB1*15:02, HLA DRB1*16:02	**0.10240558**
	6270	DVEWKFYDAQPCSDK	HLA DRB1*15:01, HLA DRB1*15:02, HLA DRB1*16:02	**0.10088207**
	6271	VEWKFYDAQPCSDKA	HLA DRB1*15:02, HLA DRB1*16:02	**0.23032418**
	6288	IEELFYSYATHSDKF	HLA DRB1*15:02	-0.42593106
	6289	EELFYSYATHSDKFT	HLA DRB1*15:02	-0.32219001
	6309	FWNCNVDRYPANSIV	HLA DRB1*15:01, HLA DRB1*15:02	**0.05024018**
	6310	WNCNVDRYPANSIVC	HLA DRB1*15:01, HLA DRB1*15:02	**0.0029701117**
	6311	NCNVDRYPANSIVCR	HLA DRB1*15:01, HLA DRB1*15:02	-0.037843374
	6356	FDKSAFVNLKQLPFF	HLA DRB1*12:02	-0.65316442
	6357	DKSAFVNLKQLPFFY	HLA DRB1*12:02	-0.57806402
	6358	KSAFVNLKQLPFFYY	HLA DRB1*12:02	-0.37678307
	6359	SAFVNLKQLPFFYYS	HLA DRB1*12:02	-0.60794426
	6364	LKQLPFFYYSDSPCE	HLA DRB1*15:02	-0.84820875
	6365	KQLPFFYYSDSPCES	HLA DRB1*15:01, HLA DRB1*15:02	-0.74682862
	6366	QLPFFYYSDSPCESH	HLA DRB1*15:01, HLA DRB1*15:02, HLA DRB1*16:02	-0.95212997
	6367	LPFFYYSDSPCESHG	HLA DRB1*15:02	-0.56713728
	6385	VSDIDYVPLKSATCI	HLA DRB1*16:02	**0.15127013**
	6386	SDIDYVPLKSATCIT	HLA DRB1*16:02	-0.071389115
	6387	DIDYVPLKSATCITR	HLA DRB1*11:01, HLA DRB1*15:02 HLA DRB1*16:02	-0.0025562925
	6388	IDYVPLKSATCITRC	HLA DRB1*16:02	-0.14859683
	6432	SLWVYKQFDTYNLWN	HLA DRB1*15:02, HLA DRB1*16:02	-0.3479125
	6433	LWVYKQFDTYNLWNT	HLA DRB1*15:02, HLA DRB1*16:02	-0.23899058
	6434	WVYKQFDTYNLWNTF	HLA DRB1*15:02	-0.83413699
	6454	LENVAFNVVNKGHFD	HLA DRB1*11:01	**0.078742337**
	6455	ENVAFNVVNKGHFDG	HLA DRB1*11:01	-0.10097832
	6456	NVAFNVVNKGHFDGQ	HLA DRB1*11:01, HLA DRB1*12:02	-0.23359534
	6472	GEVPVSIINNTVYTK	HLA DRB1*12:02	**0.2542538**
	6473	EVPVSIINNTVYTKV	HLA DRB1*12:02	**0.26756126**
	6479	INNTVYTKVDGVDVE	HLA DRB1*07:01	-0.16442255
	6480	NNTVYTKVDGVDVEL	HLA DRB1*07:01, HLA DRB1*16:02	-0.24068554
	6481	NTVYTKVDGVDVELF	HLA DRB1*07:01, HLA DRB1*16:02	-0.29433181
	6487	VDGVDVELFENKTTL	HLA DRB1*15:01	-0.04113928
	6488	DGVDVELFENKTTLP	HLA DRB1*15:01, HLA DRB1*15:02	-0.27493986
	6489	GVDVELFENKTTLPV	HLA DRB1*15:01, HLA DRB1*15:02	**0.18488388**
	6490	VDVELFENKTTLPVN	HLA DRB1*15:01	-0.16436808
	6510	WAKRNIKPVPEVKIL	HLA DRB1*07:01, HLA DRB1*12:02	**0.10731795**

	6511	AKRNIKPVPEVKILN	HLA DRB1*07:01, HLA DRB1*12:02, HLA DRB1*15:01	-0.43686594
	6512	KRNIKPVPEVKILNN	HLA DRB1*07:01, HLA DRB1*12:02, HLA DRB1*15:01	-0.42446222
	6513	RNIKPVPEVKILNNL	HLA DRB1*12:02	-0.3388033
	6516	KPVPEVKILNNLGVD	HLA DRB1*12:02, HLA DRB1*15:01, HLA DRB1*15:02, HLA DRB1*16:02	-0.092818209
	6517	PVPEVKILNNLGVDI	HLA DRB1*12:02, HLA DRB1*15:01, HLA DRB1*15:02, HLA DRB1*16:02	-0.0068847756
	6518	VPEVKILNNLGVDIA	HLA DRB1*12:02, HLA DRB1*15:01, HLA DRB1*15:02, HLA DRB1*16:02	-0.25937664
	6519	PEVKILNNLGVDIAA	HLA DRB1*12:02, HLA DRB1*15:01, HLA DRB1*15:02, HLA DRB1*16:02	-0.41267881
	6567	ICAPLTVFFDGRVDG	HLA DRB1*15:01	-0.21217939
	6568	CAPLTVFFDGRVDGQ	HLA DRB1*15:01	-0.16280804
	6569	APLTVFFDGRVDGQV	HLA DRB1*15:01	-0.048799482
	6579	VDGQVDLFRNARNGV	HLA DRB1*15:01	-0.16080756
	6623	AVKTQFNYYKKVDGV	HLA DRB1*11:01	-0.51862003
	6624	VKTQFNYYKKVDGVV	HLA DRB1*11:01	-0.44180179
	6625	KTQFNYYKKVDGVVQ	HLA DRB1*11:01	-0.58921477
	6634	VDGVVQQLPETYFTQ	HLA DRB1*12:02	-0.79229209
	6635	DGVVQQLPETYFTQS	HLA DRB1*12:02	-0.74303645
	6640	QLPETYFTQSRNLQE	HLA DRB1*07:01, HLA DRB1*11:01	-1.0412404
	6641	LPETYFTQSRNLQEF	HLA DRB1*07:01, HLA DRB1*11:01	-1.25747
	6642	PETYFTQSRNLQEFK	HLA DRB1*11:01	-0.99697172
	6643	ETYFTQSRNLQEFKP	HLA DRB1*11:01	-1.243457
	6669	AMDEFIERYKLEGYA	HLA DRB1*11:01	-0.31591546
	6670	MDEFIERYKLEGYAF	HLA DRB1*11:01	-0.5096451
	6725	STVKNYFITDAQTGS	HLA DRB1*07:01	**0.067835539**
	6726	TVKNYFITDAQTGSS	HLA DRB1*07:01, HLA DRB1*11:01, HLA DRB1*16:02	**0.087124509**
	6727	VKNYFITDAQTGSSK	HLA DRB1*07:01, HLA DRB1*11:01, HLA DRB1*16:02	-0.34742572
	6728	KNYFITDAQTGSSKC	HLA DRB1*07:01, HLA DRB1*16:02	-0.18720126
	6750	LLDDFVEIIKSQDLS	HLA DRB1*07:01, HLA DRB1*11:01, HLA DRB1*15:01, HLA DRB1*15:02, HLA DRB1*16:02	-0.27733231
	6751	LDDFVEIIKSQDLSV	HLA DRB1*07:01, HLA DRB1*11:01, HLA DRB1*12:02, HLA DRB1*15:01, HLA DRB1*15:02, HLA DRB1*16:02	-0.04841137
	6752	DDFVEIIKSQDLSVV	HLA DRB1*07:01, HLA DRB1*12:02, HLA DRB1*15:01, HLA DRB1*15:02, HLA DRB1*16:02	**0.28882695**
	6881	AVLRQWLPTGTLLVD	HLA DRB1*07:01	-0.24424824
	6882	VLRQWLPTGTLLVDS	HLA DRB1*07:01	**0.070585924**
	6883	LRQWLPTGTLLVDSD	HLA DRB1*07:01	**0.094627139**
	6884	RQWLPTGTLLVDSDL	HLA DRB1*07:01	-0.03232637
	6927	SDMYDPKTKNVTKEN	HLA DRB1*11:01	-0.67485521
	6956	QQKLALGGSVAIKIT	HLA DRB1*07:01	**0.1606486**

	6957	QKLALGGSVAIKITE	HLA DRB1*07:01	**0.22250627**
	6958	KLALGGSVAIKITEH	HLA DRB1*07:01	**0.1950708**
	6982	MGHFAWWTAFVTNVN	HLA DRB1*15:02	-0.25847532
	7016	QIDGYVMHANYIFWR	HLA DRB1*15:02	-0.44029832
	7017	IDGYVMHANYIFWRN	HLA DRB1*15:02, HLA DRB1*16:02,	-0.51529422
	7024	ANYIFWRNTNPIQLS	HLA DRB1*07:01,	-0.44210794
	7025	NYIFWRNTNPIQLSS	HLA DRB1*07:01,	-0.27952306
	7026	YIFWRNTNPIQLSSY	HLA DRB1*07:01,	-0.12275809
	7027	IFWRNTNPIQLSSYS	HLA DRB1*07:01,	-0.2564464
	7049	PLKLRGTAVMSLKEG	HLA DRB1*07:01,	-0.081985341
	7053	RGTAVMSLKEGQIND	HLA DRB1*12:02,	-0.38498524
	7054	GTAVMSLKEGQINDM	HLA DRB1*12:02,	-0.68864099
	7073	LSKGRLIIRENNRVV	HLA DRB1*15:01, HLA DRB1*15:02	**0.1768002**
	7074	SKGRLIIRENNRVVI	HLA DRB1*15:01, HLA DRB1*15:02	**0.38700762**
	7075	KGRLIIRENNRVVIS	HLA DRB1*15:01, HLA DRB1*15:02, HLA DRB1*16:02	**0.78951146**
	7076	GRLIIRENNRVVISS	HLA DRB1*15:01, HLA DRB1*15:02, HLA DRB1*16:02	**0.79853065**
	7082	ENNRVVISSDVLVNN	HLA DRB1*07:01, HLA DRB1*15:01, HLA DRB1*15:02	**0.79231845**
ORF2	24	LPPAYTNSFTRGVYY	HLA DRB1*07:01	**0.71760044**
	25	PPAYTNSFTRGVYYP	HLA DRB1*07:01	**0.38425103**
	52	QDLFLPFFSNVTWFH	HLA DRB1*15:02	-0.051186129
	57	PFFSNVTWFHAIHVS	HLA DRB1*15:01, HLA DRB1*15:02	**0.38458522**
	58	FFSNVTWFHAIHVSG	HLA DRB1*15:01, HLA DRB1*15:02, HLA DRB1*16:02	**0.17100646**
	59	FSNVTWFHAIHVSGT	HLA DRB1*15:01, HLA DRB1*15:02, HLA DRB1*16:02	**0.094599621**
	60	SNVTWFHAIHVSGTN	HLA DRB1*15:01, HLA DRB1*15:02, HLA DRB1*16:02	-0.056273567
	152	WMESEFRVYSSANNC	HLA DRB1*15:02	-0.00069338927
	153	MESEFRVYSSANNCT	HLA DRB1*15:02, HLA DRB1*16:02	-0.23974436
	154	ESEFRVYSSANNCTF	HLA DRB1*15:02, HLA DRB1*16:02	-0.53206807
	181	GKQGNFKNLREFVFK	HLA DRB1*12:02	-0.2540608
	182	KQGNFKNLREFVFKN	HLA DRB1*12:02	-0.39347618
	183	QGNFKNLREFVFKNI	HLA DRB1*12:02	-0.25063137
	197	IDGYFKIYSKHTPIN	HLA DRB1*16:02	-0.78037933
	201	FKIYSKHTPINLVRD	HLA DRB1*07:01	-0.56831252
	207	HTPINLVRDLPQGFS	HLA DRB1*12:02	-0.68623929
	230	PIGINITRFQTLLAL	HLA DRB1*15:01	-0.042411043
	231	IGINITRFQTLLALH	HLA DRB1*15:01, HLA DRB1*15:02	-0.38029989
	232	GINITRFQTLLALHR	HLA DRB1*15:01, HLA DRB1*15:02, HLA DRB1*16:02	**0.10772613**
	233	INITRFQTLLALHRS	HLA DRB1*15:01	-0.0071534627
	238	FQTLLALHRSYLTPG	HLA DRB1*12:02	**0.26071055**
	239	QTLLALHRSYLTPGD	HLA DRB1*15:01	**0.19107315**

	306	FTVEKGIYQTSNFRV	HLA DRB1*07:01	**0.70919003**
	307	TVEKGIYQTSNFRVQ	HLA DRB1*07:01, HLA DRB1*15:01, HLA DRB1*15:02, HLA DRB1*16:02	**0.45017873**
	308	VEKGIYQTSNFRVQP	HLA DRB1*07:01, HLA DRB1*15:01, HLA DRB1*15:02, HLA DRB1*16:02	**0.20827026**
	309	EKGIYQTSNFRVQPT	HLA DRB1*07:01, HLA DRB1*15:01, HLA DRB1*15:02, HLA DRB1*16:02	**0.46467579**
	310	KGIYQTSNFRVQPTE	HLA DRB1*07:01, HLA DRB1*15:01, HLA DRB1*15:02, HLA DRB1*16:02	**0.36164402**
	314	QTSNFRVQPTESIVR	HLA DRB1*15:02, HLA DRB1*16:02	**0.2289089**
	315	TSNFRVQPTESIVRF	HLA DRB1*15:02, HLA DRB1*16:02	-0.20021657
	321	QPTESIVRFPNITNL	HLA DRB1*15:01, HLA DRB1*15:02	-0.40062756
	322	PTESIVRFPNITNLC	HLA DRB1*15:01, HLA DRB1*15:02	-0.20003025
	323	TESIVRFPNITNLCP	HLA DRB1*15:01, HLA DRB1*15:02	-0.70352647
	324	ESIVRFPNITNLCPF	HLA DRB1*15:01	-0.43992195
	343	NATRFASVYAWNRKR	HLA DRB1*16:02	**0.47646374**
	344	ATRFASVYAWNRKRI	HLA DRB1*16:02	**0.5575169**
	371	SASFSTFKCYGVSPT	HLA DRB1*15:02	-0.12924041
	428	DFTGCVIAWNSNNLD	HLA DRB1*15:01, HLA DRB1*15:02	-0.19887616
	429	FTGCVIAWNSNNLDS	HLA DRB1*15:01, HLA DRB1*15:02, HLA DRB1*16:02	-0.10293405
	430	TGCVIAWNSNNLDSK	HLA DRB1*15:01, HLA DRB1*15:02, HLA DRB1*16:02	-0.19360068
	431	GCVIAWNSNNLDSKV	HLA DRB1*15:01, HLA DRB1*15:02	**0.022799683**
	445	VGGNYNYLYRLFRKS	HLA DRB1*11:01	**0.18580082**
	446	GGNYNYLYRLFRKSN	HLA DRB1*11:01	**0.37362504**
	492	LQSYGFQPTNGVGYQ	HLA DRB1*07:01	-0.045488993
	493	QSYGFQPTNGVGYQP	HLA DRB1*07:01	-0.053172582
	494	SYGFQPTNGVGYQPY	HLA DRB1*07:01	**0.18189287**
	495	YGFQPTNGVGYQPYR	HLA DRB1*07:01	-0.079340724
	500	TNGVGYQPYRVVVLS	HLA DRB1*15:02	-0.035053106
	501	NGVGYQPYRVVVLSF	HLA DRB1*15:02	-0.036467885
	502	GVGYQPYRVVVLSFE	HLA DRB1*15:02, HLA DRB1*16:02	**0.28627417**
	556	NKKFLPFQQFGRDIA	HLA DRB1*16:02	**0.15230751**
	631	PTWRVYSTGSNVFQT	HLA DRB1*07:01	**0.015274183**
	632	TWRVYSTGSNVFQTR	HLA DRB1*07:01	**0.10894371**
	680	SPRRARSVASQSIIA	HLA DRB1*07:01	**0.47297351**
	681	PRRARSVASQSIIAY	HLA DRB1*07:01	**0.67693256**
	689	SQSIIAYTMSLGAEN	HLA DRB1*07:01, HLA DRB1*15:01,	**0.10021838**
	690	QSIIAYTMSLGAENS	HLA DRB1*07:01	**0.01474098**
	712	IAIPTNFTISVTTEI	HLA DRB1*07:01	-0.019108711
	713	AIPTNFTISVTTEIL	HLA DRB1*07:01	-0.20095993
	714	IPTNFTISVTTEILP	HLA DRB1*07:01	-0.47793484

	715	PTNFTISVTTEILPV	HLA DRB1*07:01	**0.0062863218**
	716	TNFTISVTTEILPVS	HLA DRB1*07:01	-0.1175069
	776	KNTQEVFAQVKQIYK	HLA DRB1*12:02	-0.50438727
	777	NTQEVFAQVKQIYKT	HLA DRB1*12:02	-0.49230135
	778	TQEVFAQVKQIYKTP	HLA DRB1*12:02	-0.555042
	779	QEVFAQVKQIYKTPP	HLA DRB1*12:02	-0.69815401
	789	YKTPPIKDFGGFNFS	HLA DRB1*15:01, HLA DRB1*15:02	-0.44883338
	790	KTPPIKDFGGFNFSQ	HLA DRB1*15:01, HLA DRB1*15:02, HLA DRB1*16:02	-0.36848437
	791	TPPIKDFGGFNFSQI	HLA DRB1*15:01, HLA DRB1*15:02, HLA DRB1*16:02	-0.19527993
	792	PPIKDFGGFNFSQIL	HLA DRB1*15:01, HLA DRB1*15:02	-0.19573394
	797	FGGFNFSQILPDPSK	HLA DRB1*07:01	-0.4760424
	830	DAGFIKQYGDCLGDI	HLA DRB1*15:01	-0.26608933
	831	AGFIKQYGDCLGDIA	HLA DRB1*15:01	-0.22414894
	864	LLTDEMIAQYTSALL	HLA DRB1*15:01, HLA DRB1*15:02	**0.2463336**
	865	LTDEMIAQYTSALLA	HLA DRB1*15:01, HLA DRB1*15:02, HLA DRB1*16:02	**0.086392148**
	866	TDEMIAQYTSALLAG	HLA DRB1*15:01, HLA DRB1*15:02, HLA DRB1*16:02	**0.0082184443**
	867	DEMIAQYTSALLAGT	HLA DRB1*15:01, HLA DRB1*15:02, HLA DRB1*16:02	**0.15716941**
	868	EMIAQYTSALLAGTI	HLA DRB1*15:01, HLA DRB1*15:02, HLA DRB1*16:02	**0.14667354**
	869	MIAQYTSALLAGTIT	HLA DRB1*15:01	**0.04890301**
	894	LQIPFAMQMAYRFNG	HLA DRB1*12:02	-0.21947589
	911	VTQNVLYENQKLIAN	HLA DRB1*15:01	-0.27091446
	955	NAQALNTLVKQLSSN	HLA DRB1*11:01	-0.68803277
	956	AQALNTLVKQLSSNF	HLA DRB1*11:01	-0.67563689
	977	LNDILSRLDKVEAEV	HLA DRB1*11:01	-0.32644775
	978	NDILSRLDKVEAEVQ	HLA DRB1*11:01, HLA DRB1*12:02	-0.47575273
	988	EAEVQIDRLITGRLQ	HLA DRB1*12:02	-0.18209317
	989	AEVQIDRLITGRLQS	HLA DRB1*12:02	-0.083411268
	990	EVQIDRLITGRLQSL	HLA DRB1*12:02	-0.2821438
	1000	RLQSLQTYVTQQLIR	HLA DRB1*15:01, HLA DRB1*15:02	-0.18959048
	1001	LQSLQTYVTQQLIRA	HLA DRB1*15:01, HLA DRB1*15:02	-0.050704469
	1002	QSLQTYVTQQLIRAA	HLA DRB1*15:01, HLA DRB1*15:02	-0.014520542
	1014	RAAEIRASANLAATK	HLA DRB1*15:01, HLA DRB1*15:02	**0.29346657**
	1015	AAEIRASANLAATKM	HLA DRB1*15:01, HLA DRB1*15:02	-0.084387697
	1057	PHGVVFLHVTYVPAQ	HLA DRB1*12:02	**0.0027038643**
	1110	YEPQIITTDNTFVSG	HLA DRB1*07:01, HLA DRB1*15:02	-0.55271887
	1111	EPQIITTDNTFVSGN	HLA DRB1*07:01, HLA DRB1*15:02, HLA DRB1*16:02	-0.3530133
	1126	CDVVIGIVNNTVYDP	HLA DRB1*12:02, HLA DRB1*15:01, HLA DRB1*15:02	-0.38779121
	1127	DVVIGIVNNTVYDPL	HLA DRB1*15:01, HLA DRB1*15:02	-0.15210975
	1151	ELDKYFKNHTSPDVD	HLA DRB1*07:01, HLA DRB1*15:02, HLA DRB1*16:02	-0.40577462
	1152	LDKYFKNHTSPDVDL	HLA DRB1*07:01, HLA DRB1*15:02, HLA DRB1*16:02	-0.7444172

	1153	DKYFKNHTSPDVDLG	HLA DRB1*07:01, HLA DRB1*15:02, HLA DRB1*16:02	-0.4953733
ORF4	51	LVKPSFYVYSRVKNL	HLA DRB1*07:01	-0.39721306
	52	VKPSFYVYSRVKNLN	HLA DRB1*07:01, HLA DRB1*11:01	-0.46468316
	53	KPSFYVYSRVKNLNS	HLA DRB1*07:01, HLA DRB1*11:01	-0.52339177
	54	PSFYVYSRVKNLNSS	HLA DRB1*07:01	-0.35688916
	55	SFYVYSRVKNLNSSR	HLA DRB1*07:01	-0.4195908
	58	VYSRVKNLNSSRVPD	HLA DRB1*12:02, HLA DRB1*15:01, HLA DRB1*15:02, HLA DRB1*16:02	-0.18705221
	59	YSRVKNLNSSRVPDL	HLA DRB1*12:02, HLA DRB1*15:01, HLA DRB1*15:02, HLA DRB1*16:02	-0.45329667
ORF5	95	YFIASFRLFARTRSM	HLA DRB1*11:01	-0.35658933
	96	FIASFRLFARTRSMW	HLA DRB1*11:01	-0.3109081
	97	IASFRLFARTRSMWS	HLA DRB1*11:01	-0.29170845
	98	ASFRLFARTRSMWSF	HLA DRB1*11:01	-0.27314175
	109	MWSFNPETNILLNVP	HLA DRB1*15:02, HLA DRB1*16:02	-1.0296371
	144	ILRGHLRIAGHHLGR	HLA DRB1*11:01	**0.196423**
	145	LRGHLRIAGHHLGRC	HLA DRB1*11:01	**0.2459722**
	146	RGHLRIAGHHLGRCD	HLA DRB1*11:01	-0.18255298
	162	KDLPKEITVATSRTL	HLA DRB1*07:01	-0.46025371
	163	DLPKEITVATSRTLS	HLA DRB1*07:01, HLA DRB1*15:01, HLA DRB1*15:02, HLA DRB1*16:02	-0.36193098
	164	LPKEITVATSRTLSY	HLA DRB1*07:01, HLA DRB1*12:02, HLA DRB1*15:01, HLA DRB1*15:02, HLA DRB1*16:02	-0.17020992
	165	PKEITVATSRTLSYY	HLA DRB1*07:01, HLA DRB1*12:02, HLA DRB1*15:01, HLA DRB1*15:02, HLA DRB1*16:02	**0.2121047**
	166	KEITVATSRTLSYYK	HLA DRB1*07:01, HLA DRB1*15:01	-0.22983445
	175	TLSYYKLGASQRVAG	HLA DRB1*07:01, HLA DRB1*16:02	**0.16141022**
	176	LSYYKLGASQRVAGD	HLA DRB1*07:01, HLA DRB1*16:02	**0.19286477**
	177	SYYKLGASQRVAGDS	HLA DRB1*07:01	**0.1701859**
ORF9	49	TASWFTALTQHGKED	HLA DRB1*11:01	-0.079044179
	84	IGYYRRATRRIRGGD	HLA DRB1*11:01	**0.6416295**
	127	KDGIIWVATEGALNT	HLA DRB1*15:01	**0.36131254**
	129	GIIWVATEGALNTPK	HLA DRB1*07:01	**0.49614189**
	167	LPKGFYAEGSRGGSQ	HLA DRB1*11:01, HLA DRB1*16:02	-0.46909126
	168	PKGFYAEGSRGGSQA	HLA DRB1*11:01, HLA DRB1*16:02	-0.023816502
	263	TATKAYNVTQAFGRR	HLA DRB1*07:01	-0.35284528
	264	ATKAYNVTQAFGRRG	HLA DRB1*07:01, HLA DRB1*16:02	-0.41990668
	265	TKAYNVTQAFGRRGP	HLA DRB1*07:01, HLA DRB1*16:02	-0.89365915
	266	KAYNVTQAFGRRGPE	HLA DRB1*16:02	-0.76630992
	302	PQIAQFAPSASAFFG	HLA DRB1*07:01	**0.72358869**
	303	QIAQFAPSASAFFGM	HLA DRB1*07:01	**0.83655244**

	304	IAQFAPSASAFFGMS	HLA DRB1*07:01	**1.0616777**
	309	PSASAFFGMSRIGME	HLA DRB1*11:01	**0.94891009**
	310	SASAFFGMSRIGMEV	HLA DRB1*11:01	**0.79707565**
	311	ASAFFGMSRIGMEVT	HLA DRB1*11:01	**0.085634049**
	325	TPSGTWLTYTGAIKL	HLA DRB1*07:01	**0.12699857**
	326	PSGTWLTYTGAIKLD	HLA DRB1*07:01	**0.0035100729**
	327	SGTWLTYTGAIKLDD	HLA DRB1*07:01, HLA DRB1*15:02, HLA DRB1*16:02	-0.3048811
	328	GTWLTYTGAIKLDDK	HLA DRB1*07:01, HLA DRB1*15:02, HLA DRB1*16:02	-0.13745422
	329	TWLTYTGAIKLDDKD	HLA DRB1*07:01	-0.21623699
	330	WLTYTGAIKLDDKDP	HLA DRB1*07:01	-0.5483585
	345	NFKDQVILLNKHIDA	HLA DRB1*11:01, HLA DRB1*12:02	**0.1690602**
	346	FKDQVILLNKHIDAY	HLA DRB1*11:01, HLA DRB1*12:02	**0.29690561**
	347	KDQVILLNKHIDAYK	HLA DRB1*11:01, HLA DRB1*12:02	**0.28910866**
	348	DQVILLNKHIDAYKT	HLA DRB1*11:01, HLA DRB1*12:02	**0.13183953**
	351	ILLNKHIDAYKTFPP	HLA DRB1*15:01	-0.60424218
	352	LLNKHIDAYKTFPPT	HLA DRB1*15:01, HLA DRB1*15:02	-0.58679713
	353	LNKHIDAYKTFPPTE	HLA DRB1*15:01, HLA DRB1*15:02, HLA DRB1*16:02	-0.50811758
	354	NKHIDAYKTFPPTEP	HLA DRB1*15:01, HLA DRB1*15:02, HLA DRB1*16:02	-0.68356588
	355	KHIDAYKTFPPTEPK	HLA DRB1*15:01, HLA DRB1*15:02	-0.64058568
	356	HIDAYKTFPPTEPKK	HLA DRB1*15:01	-0.73433853
	387	KKQQTVTLLPAADLD	HLA DRB1*12:02	**0.097605744**
	388	KQQTVTLLPAADLDD	HLA DRB1*12:02	-0.18353366
	389	QQTVTLLPAADLDDF	HLA DRB1*12:02	-0.27974059
